# Siglec–15 promotes a feed-forward loop between TNBC cells and macrophages via the NF-κB/DcR3/CCL18/STAT3 axis

**DOI:** 10.1016/j.tranon.2026.103032

**Published:** 2026-09-18

**Authors:** Sizhang Wang, Hua Qi Wang, Hui Zhao, Ruixia Zhao, Peng Zhao, Qiang Mu

**Affiliations:** a The Second Department of General Surgery, Yangquan First People's Hospital, Yangquan, Shanxi, 045000, China; b Qingdao Medical College of Qingdao University, Qingdao, Shandong, 266042, China; c Department of Pathology, Qingdao Central Hospital, University of Health and Rehabilitation Sciences (Qingdao Central Hospital), Qingdao, Shandong, 266042, China; d Department of Molecular Pathology, Qingdao Central Hospital, University of Health and Rehabilitation Sciences (Qingdao Central Hospital), Qingdao, Shandong, 266042, China; e Department of Breast surgery, Qingdao Central Hospital, University of Health and Rehabilitation Sciences, Qingdao, Shandong, 266042, China

**Keywords:** Triple-negative breast cancer, Tumor-associated macrophages, Siglec-15, DcR3, CCL18

## Abstract

M2-like tumor-associated macrophages (TAMs), recognized as a significant risk factor in tumor progression, play a crucial role in the tumor microenvironment. While Sialic acid binding Ig like Lectin 15 (Siglec-15), an well-characterized immune-checkpoint inhibitor, has been demonstrated to impact immunotherapy response and tumor progression, its role in TMAs-mediated tumor progression remains largely unclear. In this study, we demonstrated that the high expression of Siglec-15 in triple-negative breast cancer (TNBC) acts as a pivotal driver of the interaction between tumor cells and macrophages. TNBC cells with overexpressed Siglec-15 can induce the polarization of macrophages into CC chemokine ligand 18 (CCL18)^+^ M2 macrophages and recruit macrophages. Furthermore, CCL18⁺ M2 TAMs can directly facilitate the malignant behaviors of TNBC cells. Mechanistically, they secrete CCL18, which promotes the expression of Siglec-15 via the phosphorylation of signal transducer and activator of transcription 3 (STAT3), thereby indirectly accelerating tumor progression. Molecular biochemical assays, including RNA sequencing on TNBC cells with overexpressed Siglec-15 and capillary reverse-phase liquid chromatography (LC)-tandem mass spectrometry (MS/MS) of the cell supernatant, demonstrated Siglec-15 upregulated the expression of Decoy receptor 3 (DcR3) in macrophages through Nuclear factor-kappa B (NF-κB) pathway. In summary, this study reveals a novel Siglec-15-mediated positive feed-forward loop between TNBC cells and macrophages, which relies on the NF-κB/DcR3/CCL18/STAT3 signaling axis to facilitate TNBC progression..

## Highlights

• Siglec-15 overexpression drives triple-negative breast cancer (TNBC)-macrophage crosstalk: Multi-omics analyses (single-cell RNA-seq, bulk RNA-seq, spatial transcriptomics) and experiments confirm that TNBC-derived Siglec-15 induces M2-type CCL18⁺ tumor-associated macrophage (TAM) polarization and recruitment.
• M2-type CCL18⁺ TAMs directly enhance TNBC malignant phenotypes and these TAMs secrete CCL18, which upregulates Siglec-15 expression in tumor cells via STAT3 phosphorylation, forming a pro-tumorigenic positive feedback loop.
• DcR3 is a key mediator of Siglec-15-induced macrophage polarization: Combined RNA sequencing and proteomic analysis identify Decoy receptor 3 (DcR3) as a core molecule in Siglec-15-mediated macrophage phenotypic changes, with its expression regulated by the NF-κB pathway.
• Unveiling the Siglec-15-mediated NF-κB /DcR3/CCL18/STAT3 axis: This study uncovers a novel feed-forward regulatory loop between TNBC cells and TAMs via this signaling axis, providing potential therapeutic targets for TNBC immunotherapy.

## Introduction

Globally, breast cancer constitutes nearly one-third of all tumors in females. In recent years, among adults under 50, it has emerged as the leading cause of cancer- related deaths in women [1,2]. Among the various subtypes of breast cancer, triple- negative breast cancer (TNBC) stands out. Due to the absence of estrogen receptor, progesterone receptor, and overexpression of human epidermal growth factor receptor 2 (HER2), TNBC exhibits higher and earlier incidences, as well as elevated recurrence and mortality rates compared to other breast cancer subtypes [3]. Simultaneously, TNBC is regarded as a heterogeneous group of diseases, and understanding its biological characteristics remains a challenge [4]. Therefore, it is imperative to explore and uncover its specific molecular mechanisms and targets.

Researchers have found tumor microenvironment (TME), composed of non-tumor stromal cells, poses a significant obstacle to achieving satisfactory clinical outcomes with immunotherapies [5]. As a crucial component of the TME, mature macrophages are recruited and can effectively influence the tumor progression and the efficacy of clinical chemotherapy [6].

Immune-checkpoint inhibitors, which target immune-checkpoint receptors, have been approved for the treatment of numerous cancers, including early-stage TNBC [7,8]. Sialic Acid Binding Ig Like Lectin 15 (Siglec-15), a member of the Siglec gene family with a characteristic sialic acid-binding immunoglobulin-type lectin structure and belonging to the immune-checkpoint receptors, has frequently been reported in cancer immunotherapy and osteoclast differentiation [[9], [10], [11]]. It has been demonstrated that Siglec-15 is highly expressed in tumor cells and tumor-associated macrophages (TAMs), promoting tumor progression and metastasis [12]. In breast cancer, many studies have explored the roles of Siglec-15 in breast cancer bone metastasis [13,14]. With the advancement of single-cell RNA sequencing (scRNA-seq), TAMs in the TME can be classified into sub- clusters based on marker genes, rather than being restricted to the typical M1/M2 types [[15], [16], [17]]. This approach offers new perspectives for understanding the functions and changes of TAMs in the TME. However, the role of Siglec-15 in TNBC and its relationship with TAMs remain unclear.

CC chemokine ligand 18 (CCL18), as a chemokine, can directly affect the malignant behaviors of tumor cells and influence immune cell recruitment and angiogenesis regulation [[18], [19], [20], [21], [22]]. In gastric cancer, colorectal cancer, and non-small cell lung cancer, researchers have observed the co-localization of CCL18 in macrophages within the TME [[23], [24], [25]]. Moreover, CCL18 promotes the polarization of macrophages towards the M2 type [26]. Nevertheless, there are few studies on CCL18 in breast cancer. Signal Transducer And Activator Of Transcription 3 (STAT3) has been shown to contribute to the progression of breast cancer through multiple mechanisms, as reported in several studies [[27], [28], [29]]. Additionally, CCL18 has been demonstrated to regulate the phosphorylation of STAT3 in various types of cancers [19,30,31]. However, the role of CCL18 in regulating STAT3 phosphorylation in breast cancer has been scarcely investigated.

The nuclear factor-kappa B (NF-κB) pathway plays a complex role in tumors. On one hand, it can recruit numerous cytokines to regulate the immune response [32,33]. On the other hand, the NF-κB pathway can directly promote tumor progression by controlling tumor vascularization, the epithelial-to-mesenchymal transition, and metastasis [[34], [35], [36]]. Decoy receptor 3 (DcR3), a member of the TNFR superfamily, has been found to be regulated by the NF-κB pathway, facilitating the progression of pancreatic adenocarcinoma and nasopharyngeal carcinoma [37,38]. Additionally, in many studies, DcR3 has been shown to induce the M2 polarization of macrophages [[39], [40], [41]]. Therefore, exploring the significance of changes in the NF-κB pathway and DcR3 in TNBC is of great importance.

In this study, by utilizing multiple types of datasets, including scRNA, bulk RNA sequencing, spatial transcription data, and proteome data, and conducting a series of experiments, we demonstrated that high Siglec-15 expression can promote a positive feed-forward loop between TNBC cells and TAMs via the NF-κB/DcR3/CCL18/STAT3 axis. Our findings may offer novel insights into the crosstalk mechanisms between TAM polarization and recruitment and the progression of TNBC cells.

## Materials and method

### Human tumor biopsies

TNBC tissues were intraoperatively collected from patients undergoing surgery at the First Department of Breast Surgery, Qingdao Central Hospital, University of Health and Rehabilitation Sciences.

### Single-cell RNA sequencing data collection and processing

In this study, we retrieved scRNA-seq data from the Gene Expression Omnibus (GEO) under accession number GSE161529. The dataset included tissues from 8 patients with triple - negative breast cancer (TNBC) and 13 normal breast tissue samples from normal patients. For the analysis of the transcriptomic data, we employed R (version 4.3). Initially, we imported each sample into the R environment using the CreateSeuratObject function from the Seurat package (version 4.1.0) [42]. Subsequently, we calculated the proportion of mitochondrial gene expression in each cell. Cells that met the following criteria were selected for further analysis: having >300 detected genes, a total transcript count exceeding 1000, and a mitochondrial gene proportion of <20%. To eliminate doublets in each sample, we utilized the doubletFinder_v3 function from the DoubletFinder package (version 2.0.3) with default parameters. After processing individual samples, we merged all samples into a single dataset. To correct for batch effects, we applied the RunHarmony function from the Harmony package (version 0.1.0) [43]. Ultimately, our dataset consisted of 21 samples and 82,669 cells, which were used for subsequent analyses.

### Dimension reduction and unsupervised clustering

For dimension reduction and unsupervised clustering, we employed the LogNormalize function from the Seurat package to normalize the meta dataset, setting the scale factor to 10,000. Subsequently, we identified the top 2000 features using the variance stabilizing transformation (VST) method for downstream analysis. The data were then scaled as part of the standard pre-processing linear transformation. Principal component analysis (PCA) with default parameters was applied to the scaled data for dimension reduction. We selected the top 40 informative PC dimensions and set the resolution to 1.0 for uniform manifold approximation projection (UMAP). Ultimately, a total of 32 distinct initial clusters were generated. The FindAllMarkers function from the Seurat package was employed to pinpoint the marker genes for each specific sub - cluster. The analysis was conducted with a minimum percentage (min.pct) set at 0.25, a log fold - change threshold (logfc.threshold) of 0.5, and the Wilcoxon rank - sum test (test.use = "wilcox"). The marker genes for each sub - cluster were carefully selected as follows: For epithelial cells, the marker gene is EPCAM. Immune cells are marked by CD45. Tumor cells are characterized by the marker genes EPCAM, KRT18, and KRT19. NK and T cells are marked by CD3D, CD3E, and NKG7. Macrophages can be identified using the marker genes CD14, CD68, and CD163. B cells are associated with the marker genes MS4A1, CD79A, and IGHG3. Dendritic cells are marked by CD1C and CD1E.

### Copy number variation (CNV) calling

Initially, we extracted the information regarding cell barcodes and the origin of cells, which were derived from either TNBC patient tissues or normal breast tissues. Subsequently, we designated the cells from normal breast tissues as the reference. The cut-off parameter was set at 0.1, and the minimum number of cells per gene was set to 10. Next, we used the kmeans function to cluster the CNV information of all cells into 10 clusters. Finally, the results were visualized using a heatmap.

### Clustering and annotation of cell-populations

Dimensional reduction was carried out on three distinct cell types: tumor cells, a combination of tumor cells and immune cells, and macrophages. Similar to the previous approach, a series of processing steps were performed, including extracting cells, correcting batch effects, normalization, identification of highly variable genes, scaling, dimension reduction, and discovery of marker genes. To ensure the biological significance of the sub-clusters, different resolution parameters were selected for each sub - cluster. Specifically, a resolution parameter of 1 was set for tumor cells, 1.5 for the combination of tumor cells and immune cells, and 0.3 for macrophages. As a result, the primary populations were identified as 16 for cancer cells, 31 for the combination of immune cells and tumor cells, and 7 for macrophages. Once all the marker genes of each cluster were identified, sub-clusters with highly similar expression patterns of marker genes were merged.

### Pseudotime trajectories analysis

In order to evaluate the differentiation of TNBC cell subtypes, a pseudotime trajectories analysis was conducted. The transcriptional expression of each gene in at least 5 cells were extracted. The CytoTRACE function from the CytoTRACE R package (version 0.3.3) was employed to infer the differentiation status. This was based on the previous dimensionality reduction results obtained through UMAP. Subsequently, the top 30 genes associated with the CytoTRACE score were selected, and their relationships were visualized via a bar plot. Simultaneously, the same data of TNBC cells subtypes was subjected to pseudotime trajectories analysis using Monocle package (version 2.26.0) with default parameters. Ultimately, the coordinate information of UMAP, pseudotime and state of cells were acquired and integrated into the CytoTRACE analysis.

### Cell-cell communication analysis

CellPhoneDB (version 5.0.0), a python-based tool, was employed to explore the connections between ligands and receptors using a pre-established database [44]. Similarly, sub–clusters of tumor cells and macrophages were merged and normalized. Then, the metadate and normalized RNA expression data were extracted and utilized for cell-cell communication analysis. The parameters of cell-cell communication analysis were configured as followed: a significance threshold of <0.05, a cell selection threshold of 10%, and 1000 iterations. We visualized these results using heatmaps.

### Scenic analysis

We employed Single-Cell Regulatory Network Inference and Clustering (SCENIC), a python-based tool (version 0.12.1) [45], to assess the activity of transcription factors (TFs) and regulons and reconstruct regulatory networks. Briefly, the normalized RNA expression data of tumor cell sub - clusters and macrophages were extracted. Using GRNboost2 (arboreto 0.1.5), gene regulatory networks (GRNs) were obtained. Subsequently, the human gene-motif rankings motif database (covering 10 kb around the transcription start site) was utilized, and the “ctx” function was applied to identify the enriched motifs. The “aucell” function was then used to score the regulons in each cell. Finally, the results of TFs were presented in heatmaps, while the regulons were visualized in scatter plots. By calculating the connection specificity index (CSI) between each regulon and TF, regulons were clustered into modules with a threshold set at 0.5. We also visualized the modules corresponding to cell sub-clusters to illustrate their correlations.

### Functional enrichment analysis

In the analysis of scRNA-seq data, we carried out Gene set variation analysis (GSVA). Initially, we extracted the 50 hallmark pathways from the msigdbr package database (version 7.5.1). Subsequently, we computed the average transcriptomic expression of cells. This calculated average was then utilized for the GSVA analysis. We employed the GSVA package (version 1.52.3), setting the method as “gsva”, kcdf as “Gaussian”, and mx.diff as “True”. Finally, the results were visualized by heatmaps. Regarding the bulk transcriptomic data and protein data, we conducted Gene Ontology (GO) and Encyclopedia of Genes and Genomes (KEGG) functional enrichment analyses. These analyses were performed using the online platform of Tsingke (Beijing, China), with a significance threshold set at <0.05.

### TNBC bulk transcriptomic data downloading and processing

First, we obtained gene transcriptomic data and clinical information for 1118 breast cancer samples from The Cancer Genome Atlas-Breast cancer (TCGA-BRCA) dataset. Based on the expression levels of estrogen receptor (ER), progesterone receptor (PR), and Her2, we selected 114 TNBC samples from this dataset. The data of fragments per kilobase of transcript per million fragments mapped (FPKM) type was selected. Similarly, from a collection of 1981 breast cancer samples in METABRIC (Molecular Taxonomy of Breast Cancer International Consortium) , we identified 320 TNBC samples and retrieved their corresponding gene transcriptomic data and clinical details. Additionally, we gathered gene transcriptomic data and clinical information for 107 TNBC samples from GSE58812 in the GEO database.

### Screening of DEGs

In the first place, gene transcriptomic data of 114 TNBC samples and 113 normal samples were merged. Then, differential gene expression analysis was carried out on all genes in this matrix using the limma package (version 3.56.2) [46]. In this analysis, we applied linear model fitting and Bayesian statistical processing to the dataset. This comprehensive approach enabled a more accurate assessment of gene expression differences. Finally, the DEG were selected for other analysis. The selection criteria were a p-value 〈 0.05 and |log2FC| 〉 1.

### Linking single-cell RNA sequencing data to bulk transcriptome data

To explore the prognostic implications of distinct sub-clusters of TNBC cells, we first established a threshold for marker gene selection. Specifically, genes with logFC greater than 1.0 and a p-value <0.05 were chosen as marker genes for each specific sub-cluster of TNBC cells. Subsequently, we employed the single-sample Gene Set Enrichment Analysis (ssGSEA) method, which is implemented in the GSVA package (version 1.52.3). This method was used to calculate the related cell abundances of the sub-cluster TNBC cells based on the bulk transcriptomic data corresponding to these marker genes from three databases. The scores obtained for each sample across the three databases were retained for subsequent survival analysis. This approach allows for a more in-depth understanding of the prognostic value of different TNBC cell sub-clusters [47].

### Survival analysis

To comprehensively analyze the impact of various elements on TNBC patients, Kaplan–Meier (KM) survival analysis was employed. First, the overall survival time and survival status of all TNBC patients were extracted and integrated with gene expression data or scores of different sub-clusters. Leveraging the “surv_cutpoint” function from the “survminer” R package (version 0.4.9), all samples were divided into “high” and “low” groups. Subsequently, the “survival” R package (version 3.8–3) was utilized to calculate the overall survival (OS) outcomes associated with different elements. Moreover, an OS analysis of Siglec-15 was conducted on 404 “basal” patients retrieved from the KM Plotter databases.

### Identification of prognostic genes

One-way Cox regression analysis and KM survival analysis was performed to detect the prognostic significance of genes in TNBC patients. Initially, KM survival analysis was employed to examine the interaction between T14 tumor cell marker genes and DEGs. Genes with a p-value < 0.05 were selected for subsequent in-depth analysis. Then, we utilized 10-fold cross-validation to assess 107 combinations of 11 machine learning algorithms across four datasets. The algorithms included random survival forest (RSF), ObliqueRSF, xgboost, Lasso, Ridge, CoxBoost, elastic net (Enet), supervised principal components (SuperPC), survival support vector machine (survival-SVM), CForest, and Ctree. The TCGA dataset was divided into two subsets at a 1:1 ratio. Our objective was to identify the most valuable gene model based on the highest concordance index (C-index). The predictive accuracy of the models was evaluated using receiver operating characteristic (ROC) curves and KM survival analysis. Finally, one-way Cox regression analysis was conducted on the interaction between T14 tumor cell marker genes and DEGs, and genes with a p-value < 0.05 were selected. We then chose the intersection of the significant genes identified in the KM survival analysis and the one-way Cox regression analysis for further investigation.

### Immune cell infiltration analysis and correlation analysis

To assess the association between immune cells and specific genes, an immune cell infiltration analysis was carried out. Using the “CIBERSORT” function in the R package “IOBR” (version 0.99.9), the immune scores of different immune cells for patients were calculated based on the entire gene transcriptomic data from three databases. Subsequently, a pearson's correlation test was employed to analyze the correlation between different immune cells and genes. A p-value of <0.05 was considered statistically significant.

### Spatial transcriptomics analysis

We retrieved spatial transcriptomics data from two slides of TNBC patients from the GEO database under accession number GSE218951. Initially, we applied the “SCTransform” function from the R package “Seurat” to conduct single-cell transformation on the spatial transcriptomics data, focusing on the top 3000 genes. Then, we used the “FindIntegrationAnchors” function in the “Seurat” R package to identify anchors and integrated these data. Similar to the analysis process of scRNA-seq data, we carried out dimensionality reduction and unsupervised clustering on the spatial transcriptomics data. We set the resolution parameter to 0.5 and considered the top 20 dimensions. After that, to correct for batch effects, we employed the “harmony” function from the Harmony package (version 0.1.0) with the “SCT” method. Finally, clustering and annotation of cell–populations were performed using “singleR” function by “SingleR” R package (version 2.6.0).

### Cell lines and treatments

The cell lines THP-1 monocytes (CL-0233), MDA-MB-231 (CL-0150), BT549 (CL-0041) were purchased from Procell (Wuhan, China). All TNBC cells were maintained in DMEM medium supplemented with 10% fetal bovine serum (FBS) and antibiotics (1% penicillin/streptomycin). THP-1 cells were maintained in RPMI 1640 medium supplemented with 10% FBS and antibiotics (1% penicillin/streptomycin) at 37 °C in a humidified 5% CO2 incubator. To obtain tumor cell-conditioned medium (TCM), MDA-MB-231 and BT549 cells were cultured in DMEM supplemented with 10% FBS for 48 h. In addition, macrophage-conditioned medium (MCM) was harvested from the co-culture system of M0-differentiated tumor-associated macrophages (TAMs) and TNBC cells. Both TCM and MCM were centrifuged at 1200 g for 10 min to remove cell debris, and the supernatants were stored at −80 °C for subsequent experiments. For NF-κB pathway inhibition, TNBC cells were treated with 10 μM QNZ (Selleck, #EVP4593) for 24 h.

### Polarization of THP-1 cells

To differentiate THP-1 cells into macrophages, THP-1 monocytes were incubated with 100 μg/mL Phorbol-12-myristate-13-acetate (PMA, Beyotime, S1819) for 24 h. Subsequently, 20 ng/mL of interleukin-4 (IL-4) and 20 ng/mL of interleukin-13 (IL-13) were supplemented into the culture medium for 48 h to obtain M2-type macrophages.

### Generation of stable overexpression and knockdown cancer cell lines

The full-length coding sequences of human Siglec-15 (NM_213602.3:55-1041) were subcloned into the LV5 (EF - 1a/GFP&Puro) vector to construct Siglec-15-overexpressing TNBC cell lines. For establishing stable gene knockdown in TNBC cells, short hairpin RNAs (shRNAs) were employed. Meanwhile, the full-length coding sequences of human TNFRSF6B (NM_003823.4) were cloned into the ZsGreen-PURO vector.

All constructed plasmids were thoroughly verified through DNA sequencing. Lentiviruses carrying the plasmid targeting human Siglec-15 were purchased from GenePharma company (Suzhou, China), and Lentiviruses targeting human TNFRSF6B were obtained from Hanbio company in (Shanghai, China). TNBC cells were infected with the corresponding lentiviruses in the presence of 5 μg/mL polybrene (GenePharma) and incubated for 24 h. Subsequently, puromycin (GenePharma) was used for screening to obtain stably transfected cell lines. The efficiency of gene overexpression and knockdown was confirmed by Western blotting.

### Quantitative real-time PCR (qRT-PCR)

Total RNA was isolated from cells using RNAeasy™ Animal RNA Isolation Kit with Spin Column (Beyotime, R0026), following the manufacture’s protocol. Total RNA from each sample was reversely transcribed into complementary DNA (cDNA) using HiScript III RT SuperMix for qPCR (+gDNA wiper) (vazyme, R323-01). Then, qRT-PCR were performed with ChamQ Universal SYBR qPCR Master Mix (vazyme, Q711-02). Relative gene expression levels were calculated using the method of 2^−ΔΔCt^, with GAPDH used as the internal reference gene.

### Western blotting

Cells were lysed with RIPA lysis buffer (Beyotime, P0013) containing Pheny methy sulfony fluoride (SOLARBIO, P0100) and Phosphatase inhibitor cocktail I, 100 × (Meilunbio, MB12707-1). After incubation on ice for 20 min, the mixtures were centrifuged at 10,000 g for 20 min at 4 °C. The supernatant containing proteins was harvested to measure its concentration using bicinchoninic acid (BCA) assay (SOLARBIO, PC0020), according to manufacture’s protocol. Equal 20 μg proteins were denatured by boiling at 100 °C for 5 min in sodium dodecyl sulfate‒polyacrylamide agarose gel electrophoresis (SDS-PAGE) Sample Loading Buffer (Beyotime, P0015). The mixtures were separated using 8%−12% SDS‒PAGE and transferred into PVDF membranes (Millipore, IPVH00010). Then, the membranes were incubated in the primary antibodies overnight at 4 °C. Subsequently, the membranes were incubated with HRP-labeled Goat Anti-Rabbit IgG (H+L) for 1 h, at room temperature. The protein bands were detected using Fluorescent&Chemiluminescence Gel Imaging System (Peiqing Science and Technology, China). The primary antibodies were as follows: anti-Siglec-15 (proteintech, #285941-AP, 1:10000), anti-TNFRSF6B (proteintech, #24999-1-AP, 1:1000), anti-Phospho-NF-κB p65 (proteintech, #82335-1-RR, 1:10000), anti- NF-κB p65 (Abcam, #AB32536, 1:10000), anti-Phospho-IKBα (Selleck, #A5983, 1:1000), anti-IKBα (Selleck, #A5599, 1:1000), anti-β-Actin (Selleck, #F0012, 1:10000), anti-β-Actin (Selleck, #F0012, 1:10000), anti-p-STAT3 (Selleck, #F0157, 1:2000), anti-STAT3 (proteintech, #80149-1-RR, 1:4000), anti-β-Tubulin (Selleck, #F0167, 1:10000).The following secondary antibodies were used: Anti-rabbit IgG HRP linked-antibody (Beyotime, #A0208, 1:1000). Finally, the protein bands were analyzed by software Image J (National Institutes of Health, Bethesda, USA).

### Transwell co-culture assays

To explore the regulatory effects of Siglec-15 and TNFRSF6B on macrophage polarization, a Transwell co-culture system was performed. Briefly, 3 × 10^5^ MDA-MB-231 and BT549 cells with different vectors (OE-Siglec-15, OENC, OE-Siglec-15 + SH-TNFRSF6B, OE-Siglec-15 + SHNC, OENC+ SH- TNFRSF6B, OENC+SHNC) were seeded into the upper chamber (0.4 μm pore size, 24 mm diameter; Corning Incorporated, USA). And 1 × 10^6^ M0-differentiated TAMs cells were seeded in the lower chamber. After 24 h of co-culture, the medium in the lower chamber was replaced with fresh RPMI 1640 medium containing 10% FBS, and TAMs were continuously incubated for another 48 h. Finally, TAMs were harvested for subsequent qRT‑PCR and flow cytometry analyses.

### Macrophage recruitment

To study the effect of Siglec-15 and TNFRSF6B on macrophage recruitment, 2 × 10^5^

M0 and M2-differentiated TAMs cells with 200 μL RPMI 1640 medium without FBS were seeded in the upper chamber (8.0 μm pore size, 6.5 mm diameter; Corning Incorporated, USA). 600 μL collected TCM followed by previous protocol were added into the lower chamber. After 72 h of incubation, the TAMs in upper chamber were fixed in organizational fixative (Servicebio, G1101) and detected by crystal violet. Migrated macrophages were photographed and quantified using ImageJ software.

### Flow cytometry

The M0-differentiated TAMs cells were harvested from the co-culture system to prepare into single-cell suspensions. Subsequently, these suspensions were incubated with 200 μL APC-conjugated anti-human CD163 antibodies (Abcam, #AB182422, 1:60), anti-human CD86 antibodies (Abcam, #AB239075, 1:1000) for 30 min at 4 °C. Then, they were further incubated with Goat Anti-Rabbit IgG (H+L) (Abcam, #AB150077, 1:4000) for 30 min at 4 °C. Cell phenotypic analysis was subsequently performed using a BriCyte E6 flow cytometer (Mindray, China). All acquired flow cytometry data were analyzed with FlowJo software (FlowJo, Ashland, OR, USA).

### ELISA

The TCM from MDA-MB-231 and BT549 TNBC cells (OE-Siglec-15, OENC) were analyzed using the human TNFRSF6B ELISA KIT (Mlbio, ml038582), following the protocol of manufacturer. The levels of TNFRSF6B were measured at 450 nm by microplate reader (Huisong, #MB-530).

### Cell viability cell counting kit-8 (CCK-8) assay

5 × 10^3^ MDA-MB-231 and BT549 TNBC cells were seeded into a 96-well plate and treated with 100 μL MCM and cultured at 37 °C. 10 μL of CCK-8 reagent (Solarbio, CA1210) were added into each plate at 0, 24, 48, 78 h and the cells were further incubated for 1 h at 37 °C. Then, the absorbance values of cells were detected at 450 nm by microplate reader.

### Wound healing assay

TNBC cells were initially seeded in a 6-well plate containing 2 mL of DMEM medium supplemented with 10% FBS. The plates were then incubated at 37 °C for 1 day. Once the cells reached 90% confluence, a 200-μL pipette tip was used to create a scratch on the cell monolayer. Subsequently, 2 mL of a 1:1 mixture of RPMI 1640 medium without FBS and MCM was added to the wells to facilitate cell growth over a 24-hour period. Photographs of the scratched areas were captured at appropriate time points (0 h and 24 h). The relative wound width of the TNBC cells was measured using ImageJ software.

### Cell migration and invasion assay

Transwell assay was carried out to evaluate the migration and invasion capabilities of MDA - MB - 231 and BT549 TNBC cells cultured with different MCMs. For cell migration assay, 8 × 10^5^ TNBC cells were resuspended in 200 μL of RPMI 1640 medium without FBS and then seeded into the upper transwell chamber (8.0 μm pore size, 6.5 mm diameter; Corning Incorporated, USA). Simultaneously, 600 μL of MCM was added to the lower chamber. After 24 h of incubation at 37 °C, cells that migrated to the lower surface of the membrane were fixed, stained, and photographed following the aforementioned standard protocol.

For the invasion assay, the upper chamber was first pre-coated with 100 μL of Matrigel (Corning, New York, USA, #354,234) (diluted 1:8 with RPMI-1640). And the chambers were incubated at 37 °C for 4 h. Subsequent experimental procedures were consistent with those of the migration assay.

Capillary Reverse - Phase Liquid Chromatography (LC)-Tandem Mass Spectrometry (MS/MS) of the Cell Supernatant and Data Analysis

Secretory proteins from transfected BT549 cells were identified via LC-MS/MS. Briefly, cells at 80% confluence were cultured in serum-free RPMI 1640 medium for 48 h. Cell supernatants were collected, centrifuged at 1200 g for 10 min to remove debris, and concentrated to 5 mL by lyophilization. After supplementation with 1 mM PMSF, samples were centrifuged at 4500 g for 10 min at 4 °C, and total protein concentrations were measured using a BCA assay. A total of 100 μg of protein from each sample was digested with trypsin overnight at 37 °C. The obtained peptides were pH-adjusted, desalted with C18 resin (Millipore, Billerica, MA, USA), and separated using a Vanquish Neo UHPLC system. Mass spectrometry analysis was performed in data-independent acquisition (DIA) mode on an Orbitrap Astral high-resolution mass spectrometer (Thermo Scientific). Raw data were analyzed with DIA-NN (v1.8.1) using a library-free approach with a 1% false discovery rate (FDR) threshold at both the protein and precursor ion levels for subsequent quantitative analysis.

### Cell RNA sequencing (RNA-seq) and data analysis

To conduct a comprehensive analysis of the transcriptomic data from transfected BT549 cells, cell RNA sequencing was carried out. Briefly, cells at 80% confluence were digested with trypsin and centrifuged at 1200 g for 5 min, and the supernatant was discarded. Cell pellets were lysed with 1 mL TRIzol reagent (Thermo Scientific, 15596026CN). RNA concentration and integrity were assessed using Nanodrop 2000, Agilent 2100, and LabChip GX (Thermo Scientific). Purified mRNA was enriched using mRNA Capture Beads (Thermo Scientific, N411–03) with repeated binding and washing steps. The qualified mRNA was fragmented, reverse-transcribed into cDNA, and amplified. RNA-seq libraries were constructed using the VAHTS Universal V6 RNA-seq Library Prep Kit for Illumina (Vazyme, NR604–02). After quality validation with a Qsep-400 analyzer, libraries were sequenced on the Illumina NovaSeq X Plus platform. Raw sequencing data were trimmed, aligned to the human reference genome (Ensembl v96), assembled, and quantified to generate FPKM expression profiles for subsequent analysis.

### Animal experiment

Four- to five-week-old BALB/c nude mice were purchased from Qingdao QinDa Biotechnology (Qingdao, China). All animal experimental procedures were approved by Ethics Committee of the School of Bioengineering of Qingdao Technology University. Mice were raised under specific pathogen-free (SPF) conditions with a standardized healthy environment. To explore the effect of Siglec-15 on TNBC tumor growth, stably transfected MDA-MB-231 cells (empty vector and Siglec-15 overexpression groups) were resuspended in 100 μL of PBS–Matrigel mixture (1:1 ratio) at a density of 5 × 10^6^ cells per mouse and subcutaneously injected into the mouse armpit. Tumor volume was monitored every three days throughout the experiment. All mice were humanely euthanized 30 days after injection, and xenograft tumors were harvested for the measurement of tumor weight and volume. Tumor volume was calculated using the formula: (width × length)/2 (mm).

### Statistical analysis

Statistical analyses were carried out using GraphPad Prism 8.0.2 or R 4.3.1 software. The statistical outcomes are presented as the mean values ± standard error of the mean (SEMs). Student's *t*-test or one-way ANOVA was employed to assess the significance between different groups. (In all figures: *,p < 0.05; **,p < 0.01; ***,p < 0.001; ****,p < 0.0001; ns = not significant).

## Result

### Cellular stratification of TNBC scRNA-seq data and prognostic insights into TNBC tumor cells

First, scRNA-seq data was downloaded from GSE161529 in GEO database. After removing the low quality cells and potential doublets, a total of 82,669 cells of 8 TNBC patients and 13 normal patients were collected for further analysis. To identify the major cell clusters, unsupervised clustering and UMAP plot were conducted (Supplementary Fig. 1A). Using inferCNV algorithm [48], tumor cells in TNBC were selected from all epithelial cells (marked by EPCAM, KRT8, KRT19, Supplementary Fig. 1B–F). To explore the tumor microenvironment (TME) features of TNBC, immune cells (marked by CD3E, CD45, CD68) were combined with tumor cells in TNBC patients (Supplementary Fig. 1G–I, Supplementary Fig. 2A–C). These cells were clustered into five major subsets (Fig. 1A and B, Supplementary Fig. 2A–C): tumor cells (marked by EPCAM, KRT18, KRT19), T/NK cells (marked by CD3D, CD3E, NKG7). Macrophages (marked by CD14, CD68, CD163), B cells (marked by MS4A1, CD79A, IGHG3), Dendritic cells (DCs) (marked by CD1C, CD1E). Then, 4033 TNBC tumor cells were clustered into 16 sub-populations (Fig. 1C and D). GSVA was carried out to analyze the functions of tumor cells sub-populations in more detail. The T14 tumor sub-cluster was associated with multiple metabolic processes, such as peroxisome, adipogenesis, fatty acid metabolism, bile acid metabolism, xenobiotic metabolism, cholesterol homeostasis as well as various tumor – related behaviors, including angiogenesis and IL-2 STAT5 SIGNALING (Fig. 1E). To uncover the prognostic implications of TNBC tumor cells sub-populations, the cell relative abundances of tumor cells was calculated in three datasets based on the marker genes (Log2FC > 1 and adjusted p < 0.05) of sub-populations by the way of ssGSEA algorithm. Then, KM survival analysis was performed on the score of TNBC tumor cells sub-populations. The T14 sub-cluster showed a significant negative correlation with the OS of TNBC patients in all three database, suggesting that this sub-population may play a crucial role in TNBC patients (Fig. 1F and G).

**Fig. 1:**
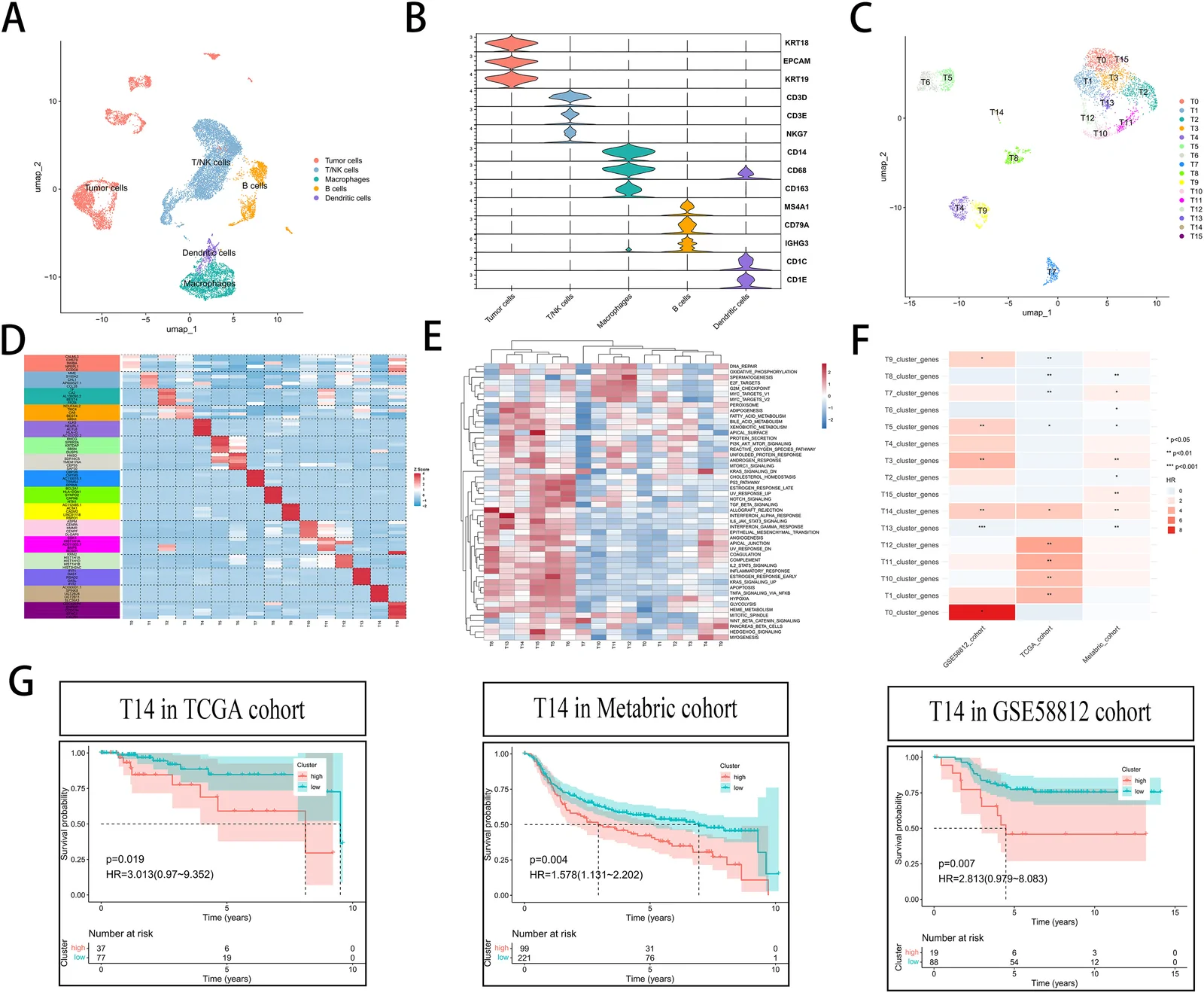
Identification of major cell types in scRNA-seq of TNBC cells and exploration of prognostic tumor cells sub-clusters. (A) UMAP plot depicting 5 major clusters in immune cells and tumor cells in TNBC patients (Tumor cells, T/NK cells, Macrophages, B cells and Dendritic cells). (B) Violin plot presenting the expression of marker genes in 5 major clusters. (C) UMAP plot showing 16 sub-clusters in tumor cells in TNBC patients. (D) Heatmap displaying the top5 expression marker genes for each sub-cluster. (E). Gene set variation analysis conducted on tumor cells sub-clusters. (F). Heatmap showing the hazard ratio value of tumor cells sub-clusters in three datasets using ssGSEA algorithm. (G). Kaplan-Meier (KM) survival analysis, which indicated that the T14 tumor cell sub-cluster was associated with a poorer prognosis in three datasets.

### Transcriptional heterogeneity of tumor cell clusters

Subsequently, we focus on the transcriptional heterogeneity of TNBC tumor cells sub-populations to realize more on these cells. CytoTRACE and Monocle2 were carried out to predict the differentiation of the tumor cells status (Fig. 2A and B, Supplementary Fig. 2D–F). We selected top30 genes, which have most correlation with the differentiation of the tumor cells (Supplementary Fig. 3A). All tumor cells were one breach break into two breaches gradually, the location of T14 sub-cluster was in the intersection of all cells. These cells may induce the differentiation of TNBC tumor cells. In addition, we analyzed the DEGs in the point of intersection and visualized most lowest 100 q-val genes through heatmap. (Fig. 2C). Among these genes, many genes, such as APOD [49], TACSTD2 [50], S100A1 [51], have been proved to influence the prognostic significance of breast cancer patients. Then, SCENIC analysis was carried out to predict the possible TFs and regulon for each cell sub-cluster using AUCell algorithm (AUC score). And we calculated Relative Specificity Score (RSS) for each sub-cluster through the score of AUC by the way of calcRSS. Through heatmap, we visualized RSS >0.1 in at least one sub-cluster (Fig. 2D). And TFs of top5 RSS value were selected (Fig. 2E). For the T14 sub-cluster, KLF13 [52], XBP1 [53] have been proven to promotes breast cancer. By setting CSI score as >0.5, 638 GRNs were identified and clustered into 4 major modules. The relationship between 4 modules and the TNBC tumor cells were identified (Fig. 2F). And top10 highest CSI GRNs were showed on the right side (Fig. 2G).

**Fig. 2:**
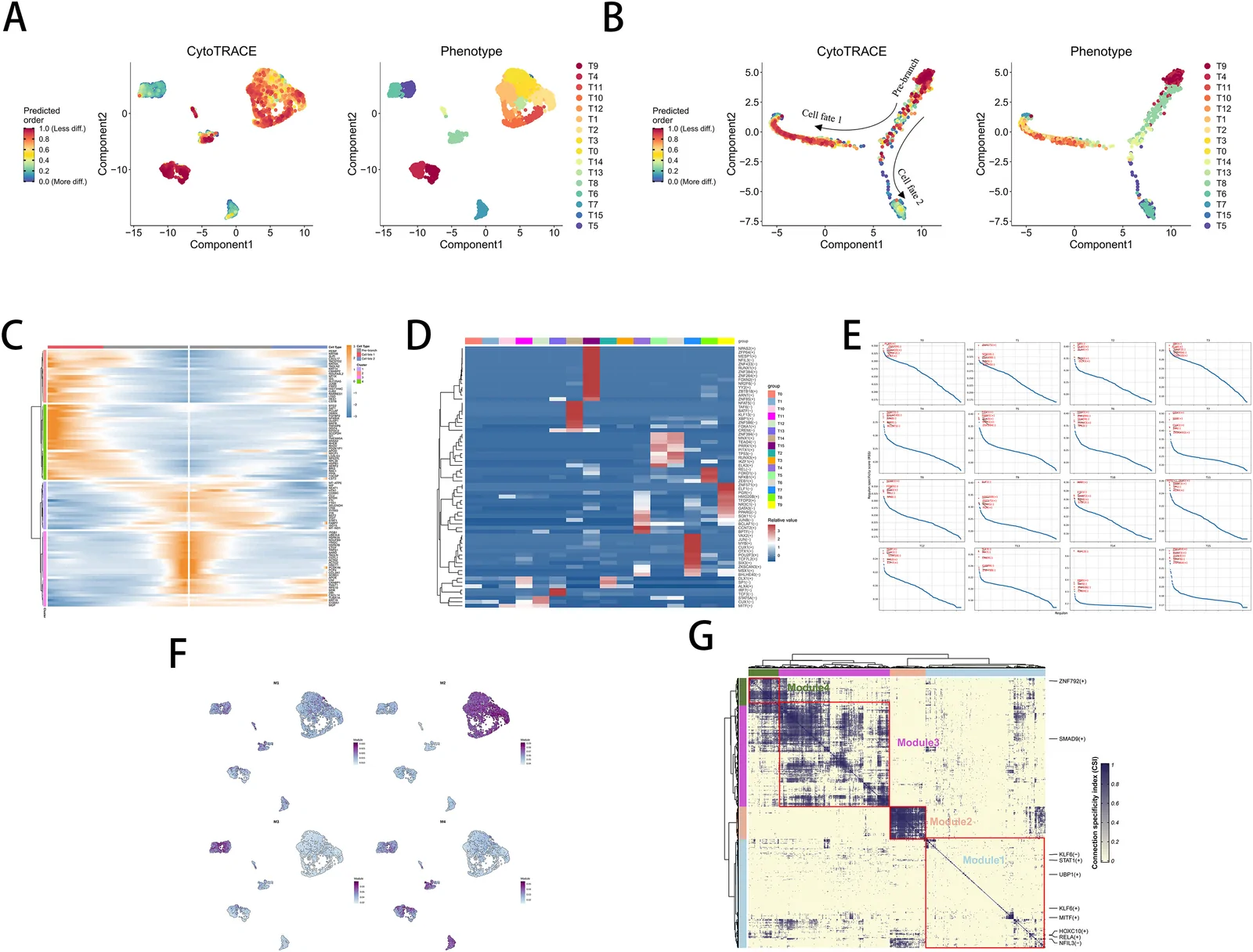
Transcriptional heterogeneity of tumor cell clusters in TNBC. (A-B) UMAP visualization and trajectory inference plots of the tumor cells, generated via CytoTRACE and Monocle2 analyses to predict tumor cell differentiation status. (C) Heatmap of the 100 genes with the lowest q-values in the intersection of all cells identified through trajectory inference. (D) Heatmap of potential transcription factors (TFs) and regulons across tumor cell sub-clusters, with values indicating the regulatory score (RSS). (E) Top 5 TFs and regulons for each sub-cluster, ranked by RSS values. (F-G) 638 gene regulatory networks (GRNs) in all tumor cells were clustered in 4 major modules; the right panel displays the top 10 GRNs with the highest connection specificity index (CSI).

### Construction and validation of an prognosis model for T14 sub-cluster

As long as we knew the significance of T14 sub-cluster TNBC tumor cells, the 437 interaction genes of the marker genes and DEGs between TNBC patients and normal patients were selected (Supplementary Fig. 3B). Then, 437 genes were performed KM survival analysis to get 149 prognostic genes (Supplementary Table1). These prognostic genes were input to 107 combinations of 11 machine learning algorithms to pick the best performance model across the score of C - index (Fig. 3A). Finally, the obliqueRSF model showed more credible than other models. All survival analysis for this model in four datasets indicated that this model was positively correlated with poorer survival in TNBC patients (Fig. 3B–E). 1/3/5 years ROC values for this model were >0.45, which was considered as a high reliability (Fig. 3F–I). Then, One-way Cox regression analysis was carried out in 437 interaction genes to choose 27 prognostic genes (Fig. 3J). In both the KM survival analysis and the univariate Cox regression analysis, Sigelec-15 showed a positive correlation with poor disease progression (Supplementary Fig. 3C).

**Fig. 3:**
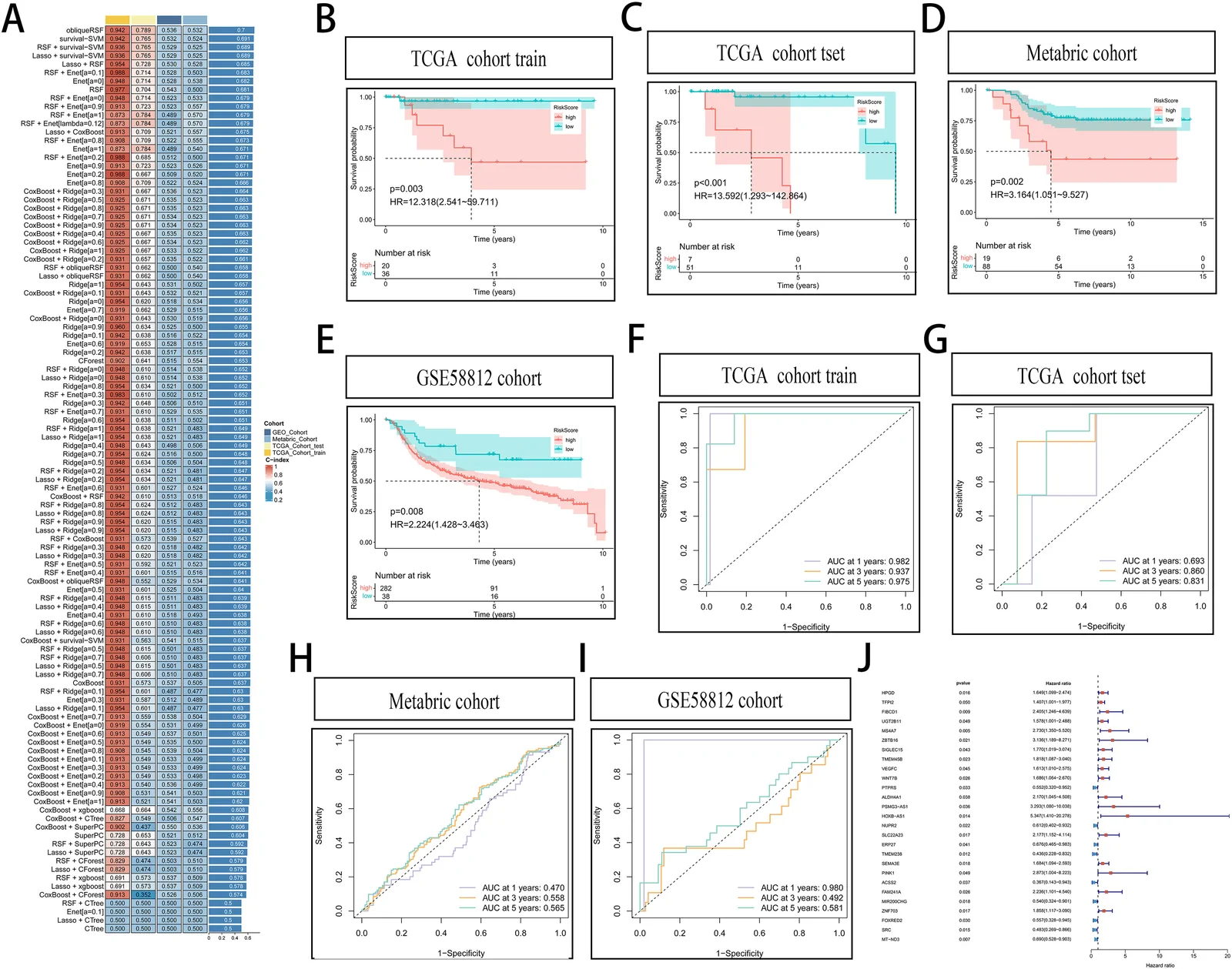
Construction and validation of survival models in T14 TNBC tumor cells sub-cluster using machine learning. (A) Construction of 107 combinations survival models employing 11 machine-learning algorithms. In the heatmap, the values represent the concordance index (C - index) of the corresponding survival models. (B-E) KM survival analysis of obliqueRSF model across four datasets. The results demonstrated that the model score was associated with a poorer disease progression. (F-I) 1/3/5 years ROC values for this model in four datasets were all greater than 0.45. (J) A forest plot from a univariate Cox regression analysis, which identified 27 prognostic genes.

### Siglec-15 may trigger the recruitment and polarization of TAMs

As widely recognized, TME plays a crucial part in tumor progression. Therefore, the interactions between TNBC cells and immune cells were analyzed by Cellphone DB analysis. Among all interactions, the connections between Macrophages and DCs cells were the most prominent, and among tumor-related cell interactions, those between macrophages and tumor cells were the most frequent (Fig. 4A). This finding suggests that Macrophages may have a significant impact on TNBC cells. Consequently, we conducted an in-depth analysis of TAMs in TNBC cells. All TAMs were classified into six clusters: Solute carrier family 40 member 1 (SLC40A1)^+^ macrophages, Versican (VCAN)^+^macrophages, Amphiregulin (AREG)^+^ macrophages, Apolipoprotein C1 (APOC1)^+^macrophages, Chemokine C—C motif ligand 18 (CCL18)^+^ macrophages, and Marker Of Proliferation Ki-67 (MKI67)^+^ macrophages (Fig. 4B, Supplementary Fig. 3D). GSVA was employed to explore the functions of TAM sub-populations. We discovered that numerous tumor-related signaling pathways, such as the PI3K-AKT-mTOR signaling pathway, the WNT-β-catenin signaling pathway, the p53 signaling pathway, and the IL6-JAK-STAT3 signaling pathway, were positively associated with CCL18^+^ macrophages (Fig. 4C). Classically, TAMs could be divided into M0/M1/M2 type macrophages, with M2 type macrophages generally regarded as pro-tumorigenic. We used M2 markers and immune markers to score all macrophage sub–clusters. CCL18^+^macrophages obtained high score for both M2 markers and immune markers (Fig. 4D, Supplementary Fig. 3E and F). Then, we analyzed the interactions between tumor cells sub-clusters and macrophages sub-clusters. VCAN^+^macrophages have the most extensive connections with T14 tumor cells, and the interaction between APP and CD74 showed highest expression level (Fig. 4E and F, Supplementary Fig. 3G). Meanwhile, CytoTRACE and Monocle2 were utillized to predict the differentiation of the TAMs status (Fig. 4G, Supplementary Fig. 3H–I). MKI67^+^ macrophages were found in the initial state, while VCAN+ macrophages were in the final state. To more comprehensively validate the characteristics of M2 type TAMs of CCL18^+^macrophages, the spital transcription data of two TNBC tissue slides were gathered (Supplementary Fig. 4A and B). Following unsupervised clustering and cell annotation of cell population by “singleR” algorithm, all cells were classified into 3 types: fibroblasts, macrophages and epithelial cells. The M2 type TAMs markers (CCL2, CD163) exhibited expression patterns consistent with that of CCL18 in macrophages (Fig. 4H and I). Then, in order to analyze the specific mechanism, we performed immune cell infiltration analysis on the T14 marker genes with significant prognostic implications from KM analysis using the “CIBERSORT” (Fig. 4J). We could find that Sialic Acid Binding Ig Like Lectin 15 (Siglec-15) was positively correlated with M0/M2 TAMs and negatively correlated with M1 TAMs (Fig. 4J and K, Supplementary Fig. 4C–E). Simultaneously, Siglec-15 showed a significant negative correlation with the OS of TNBC patients in TCGA, GEO and KM databases (Fig. 4L, Supplementary Fig. 4F). These results lead us to hypothesize Siglec-15 plays a vital role in the crosstalk between tumor cells and TAMs in TNBC cells.

**Fig. 4:**
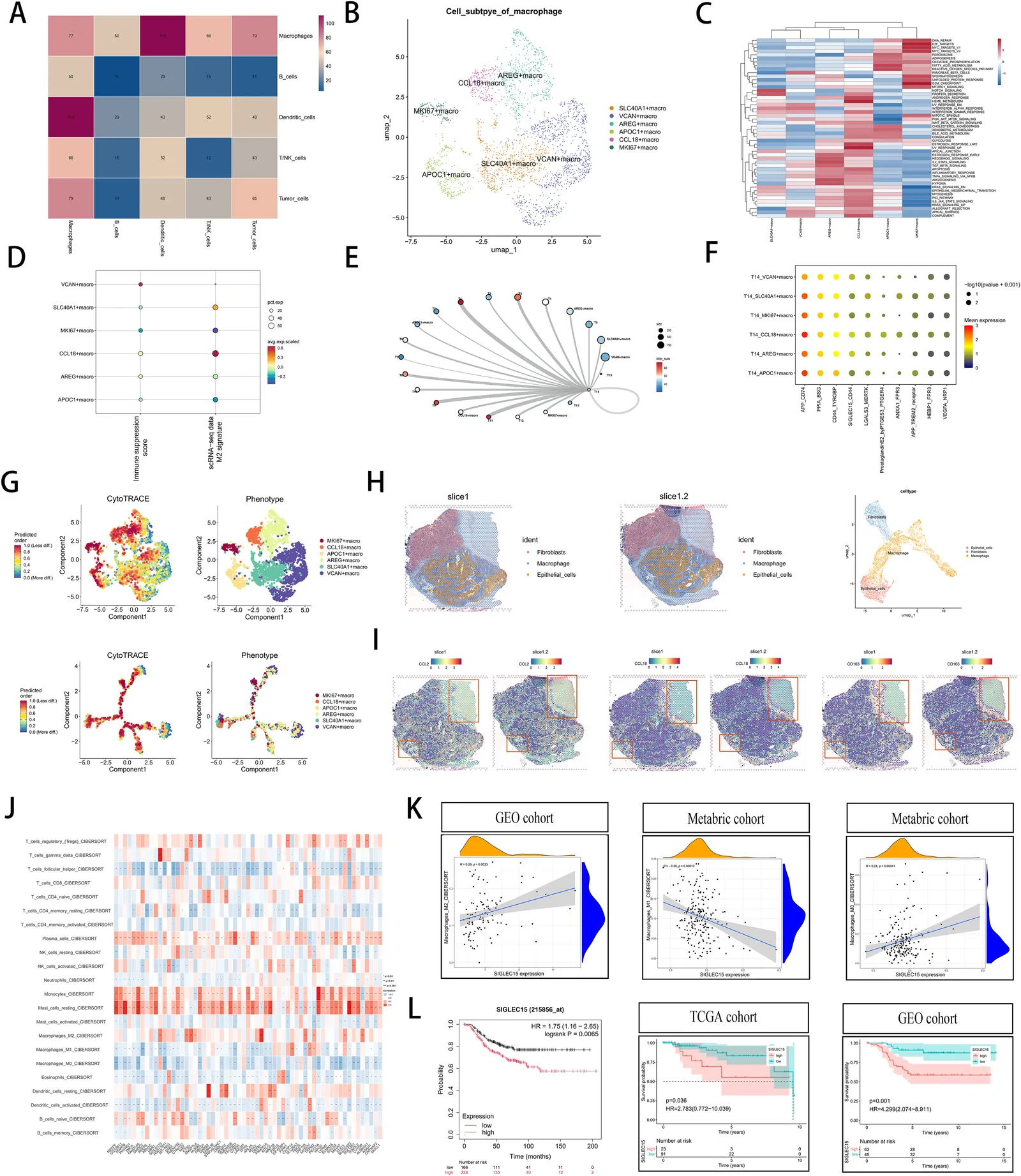
Dissection of macrophages in TNBC and the functional characteristics of Siglec-15. (A) Heatmap of tumor cell interactions with other immune cells, with values representing the number of interactions as determined by Cellphone DB analysis. (B) UMAP visualization of macrophage clusters in TNBC patients, identifying 6 major subsets: Solute carrier family 40 member 1 (SLC40A1)^+^macrophages, Versican (VCAN)^+^macrophages, Amphiregulin (AREG)^+^ macrophages, Apolipoprotein C1 (APOC1)^+^macrophages, Chemokine C—C motif ligand 18 (CCL18)^+^ macrophages, and Marker Of Proliferation Ki-67 (MKI67)^+^ macrophages. (C) Gene set variation analysis (GSVA) of macrophage sub-clusters, revealing functional pathway enrichment differences. (D) Dot plot of immune suppression scores and M2 polarization signatures scores across the 6 macrophage sub-clusters in TNBC tumor cells. (E) Dot plot quantifying interactions between tumor cell sub-clusters and macrophage sub-clusters, with size/color indicating interaction frequency. (F) Dot plot highlighting the significant interactions between T14 tumor cell sub-cluster and macrophages sub-clusters. (G) UMAP and trajectory inference plots of the macrophages, generated via CytoTRACE and Monocle2 analyses to predict macrophage differentiation status. (H) Slice plots and UMAP visualization of three major sub-clusters (fibroblasts, macrophages and epithelial cells) in two TNBC spatial transcriptomics slices. (I) Expression patterns of M2 marker genes (CCL2, CCL18, CD163) in two TNBC spatial transcriptomics slices. (J) Heatmap of correlations between 22 immune cell infiltration levels and T14 marker genes with significant prognostic value (validated by Kaplan-Meier (KM) analysis). (K) Scatter plots displaying Siglec-15 expression correlations: positive association with M0/M2 macrophages and negative association with M1 macrophages. (L) KM analysis indicating that higher Siglec-15 expression correlates with poorer overall survival in TNBC patients.

### High expression of Siglec-15 could promote the recruitment and polarization of TAMs

Initially, tissues from 6 TNBC patients were collected and digested into protein to detect the expression of Siglec-15. Trough Western blotting analysis, it was found that the expression of Siglec-15 in tumor tissues was higher than that in the matched adjacent para - carcinoma tissues (Fig. 5A).

**Fig. 5:**
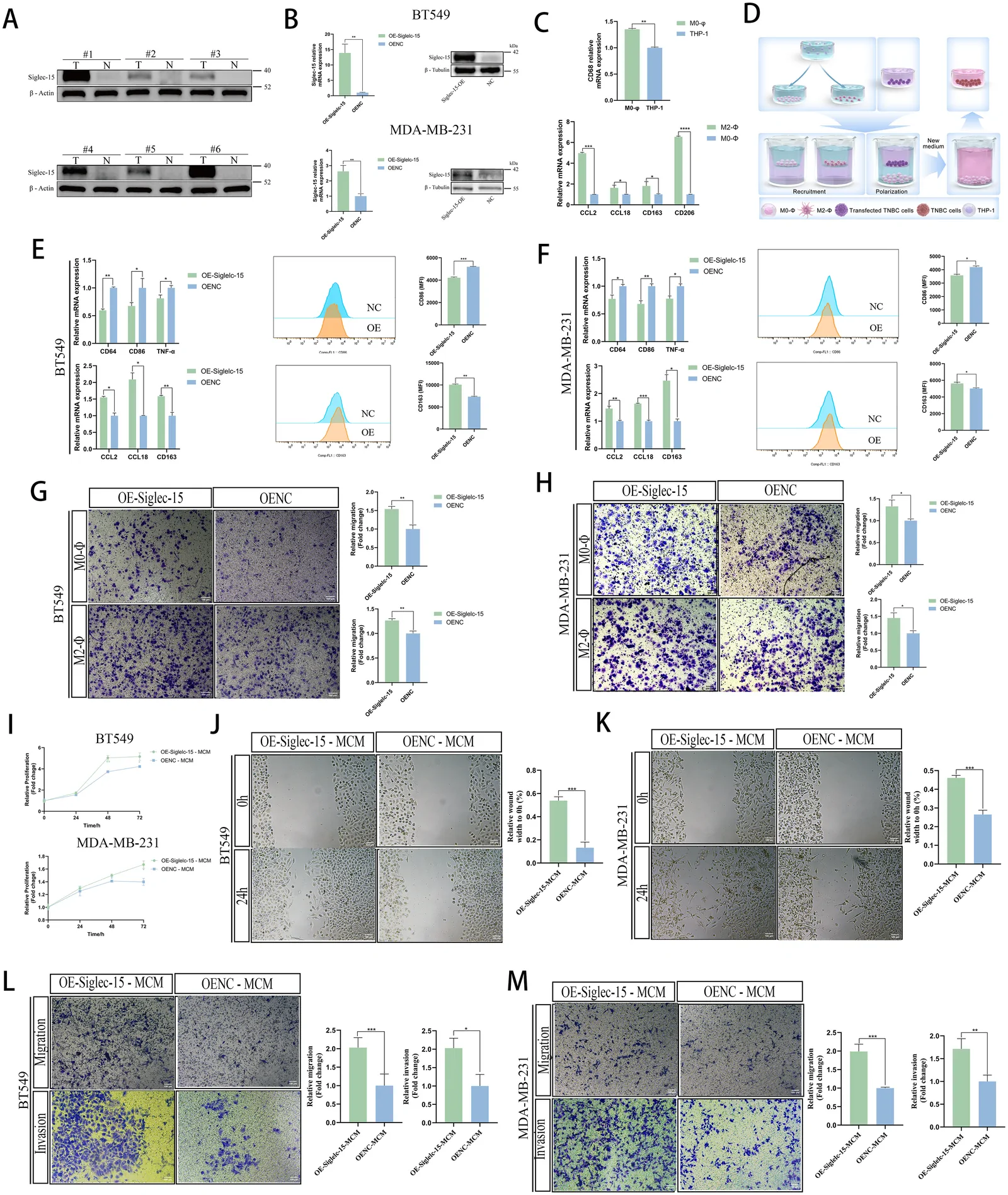
High Siglec-15 expression promote the interactions between TNBC cells and macrophages. (A) Western blotting analysis comparing Siglec-15 protein levels between TNBC patients tissues (T) and matched adjacent para-carcinoma (N) from patients. (B) Validation of Siglec-15 overexpression in TNBC cell lines via Quantitative real-time PCR (qRT‒PCR) (mRNA) and Western blotting (protein), using vector-transfected cells as controls. (C) qRT‒PCR quantification of marker gene expression in M0 macrophages (M0-φ) and M2 macrophages (M2-φ). (D) Schematic diagram illustrating the in vitro co-culture model designed to study the interactions between TNBC tumor cells and macrophages. (E-F) Siglec-15 induces the polarization of M0 macrophages to M2 type-CCL18⁺ macrophages by qRT‒PCR and flow cytometry. (G-H) Siglec-15-overexpressing TNBC cells promote the recruitment of both M0 and M2 macrophages (quantified by cell count in lower chambers) via transwell migration assays. (I) The conditional medium from co-culture system of M0-differentiated tumor-associated macrophages (TAMs) and MDA-MB-231, BT549 TNBC cells with differentially expressed genes (MCM) of overexpressing Siglec-15 system significantly enhances TNBC cell proliferation compared to control MCM by viability CCK-8 cell viability assay. (J-K) Wound-healing assay revealing accelerated closure of scratch wounds in TNBC cells treated with MCM from Siglec-15-overexpressing co-cultures system (quantified by wound width reduction over time). (L-M) Transwell invasion/migration assays demonstrating increased penetration of TNBC cells through Matrigel (invasion) or uncoated filters (migration) when treated with MCM from Siglec-15-overexpressing co-cultures system. (*p < 0.05; **p < 0.01; ***p < 0.001; ****p < 0.0001).

Then, a lentivirus expression system was employed to generate Siglec-15 or an empty vector in BT549 and MDA-MB-231 TNBC cells, establishing a stable control system and a Siglec-15 overexpression system. These cell lines were used to explore the underlying molecular mechanisms induced by Siglec-15. The transfection efficiency was verified by qRT-PCR and Western blotting (Fig. 5B).

M0, M2 macrophages were induced by PMA and IL-4, IL-13. The identities of these cells were confirmed by their specific biomarkers (CD68, CCL2, CCL18, CD163, CD206) using qRT - PCR (Fig. 5C). After that, a co-culture system was constructed to analyze the role of Siglec-15 in the recruitment and polarization of TAMs (Fig. 5D). Meanwhile, the TCM and MCM were collected from the co-culture system. Then, THP-1 derived M0 macrophages were co-cultured with Siglec-15 overexpressing TNBC cells to determine the influence of Siglec-15 on TAMs polarization. By evaluating the typical M1 macrophages biomarkers (CD64, CD86, TNF-α) and M2 macrophages biomarkers (CCL2, CCL18, CD163) via qRT-PCR and flow cytometry (Fig. 5E and F), it was found that in the co-culture system, as the expression of Siglec-15 increased, the levels of M2 biomarkers in THP-1-derived M0 macrophages increased, while those of M1 biomarkers decreased. These results indicated that Siglec-15 could induce the polarization of M0 TAMs to M2 type-CCL18⁺ TAMs. Simultaneously, a transwell assay was conducted to assess the effect of Siglec-15 on the recruitment of M0 and M2 TAMs. The number of M0 and M2 TAMs that passed through the upper chamber in the Siglec-15 overexpression TCM group was greater than that in the control group (Fig. 5G and H).

### Siglec-15 could promotes the malignant behaviors of TNBC cells through M2 type- CCL18+TAMs

MCM collected from the TAM polarization system was added to wild - type TNBC cells to investigate whether Siglec-15 could influence the malignant behaviors of TNBC cells by inducing changes in TAMs. A CCK-8 assay was carried out, and the results showed that MCM from Siglec-15 overexpressing conditions could promote the proliferation of TNBC cells (Fig. 5I). Moreover, a wound-healing assay and a transwell assay were employed. The wound - healing assay and transwell assay results indicated that MCM from Siglec-15 overexpressing conditions could enhance the migration and invasion abilities of TNBC cells (Fig. 5J–M).

### Siglec-15 was regulated by CCL18 via STAT3 phosphorylation

Furthermore, we investigated whether M2 TAMs could promotes the expression of Siglec - 15 in TNBC cells. The CM of M0/M2 TAMs were collected and added into wild type TNBC cells. By qRT-PCR, we discovered that the expression of siglec-15 in the CM of M2 TAMs was higher than in the CM of M0 TAMs (Fig. 6A). In order to explore the mechanism underlying the upregulation of Siglec-15 by M2 TAMs, the Database of Human Transcription Factor Targets (hTFtargets) was employed to predict 21 transcription factors (TFs) of Siglec-15. Subsequently, these 21 potential TFs were input into the JASPAR database to predict the binding sites between these TFs and the Siglec-15 promoter. Among them, Signal Transducer And Activator Of Transcription 3 (STAT3) achieved a high relative score, indicating that it might be the most crucial TF for the regulation of Siglec-15 (Fig. 6B). When external CCL18 was added, the expression of siglec-15 and the phosphorylation level of STAT3 were found to increase (Fig. 6C–E). These results suggest that CCL18 can promote the phosphorylation of the transcription factor STAT3, thereby enhancing the expression of Siglec-15.

**Fig. 6:**
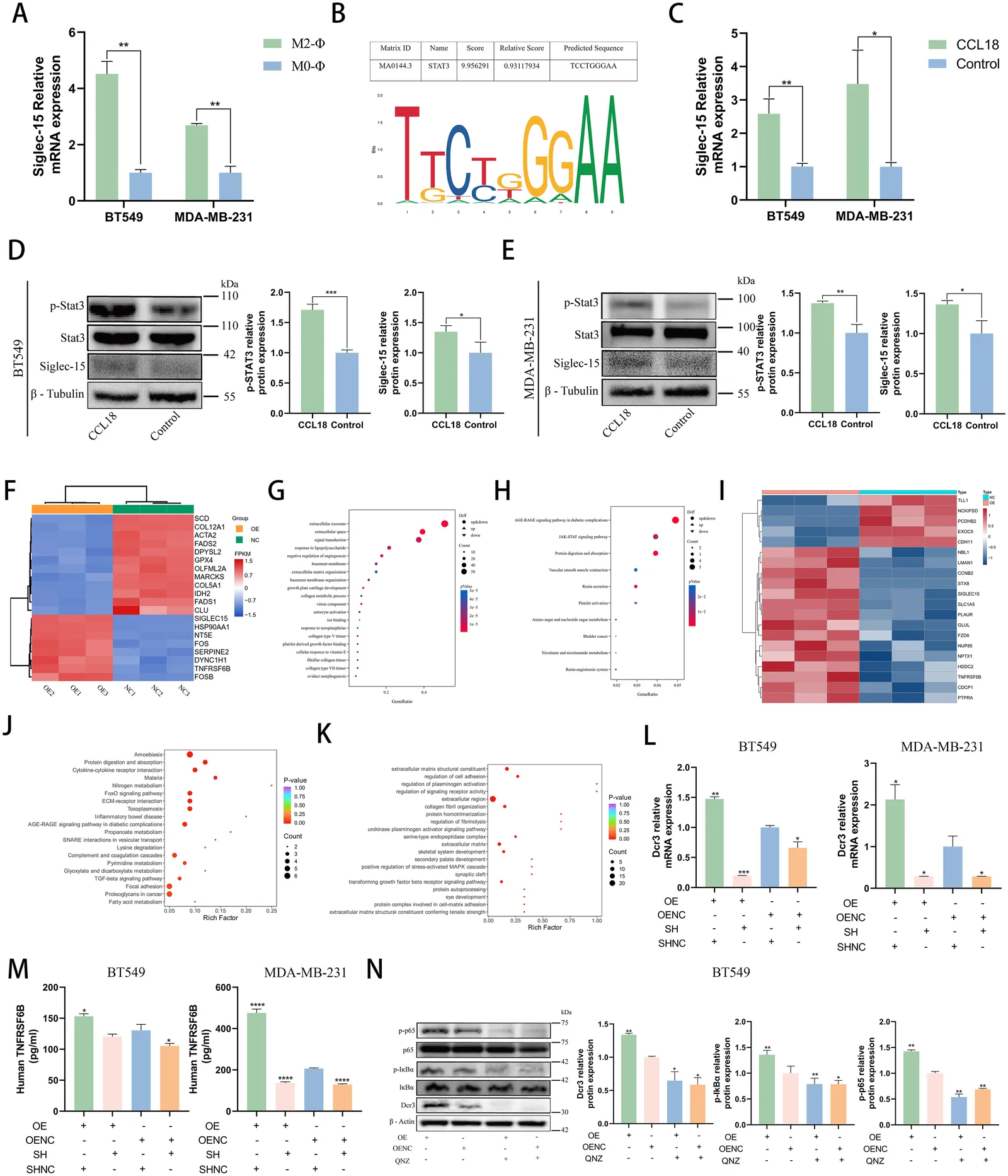
CCL18 promote Siglec-15 expression via transcription factor STAT3 phosphorylation and Siglec-15 upregulates DcR3 through NF-κB Pathway activation (A) quantitative real-time PCR (qRT‒PCR) demonstrated that conditional media from M0 and M2 macrophages (M0-φ and M2-φ) promote Siglec-15 expression. (B) The binding site of STAT3 on Siglec-15 promoters was predicted using the JASPAR database. (C) qRT-PCR confirmed that CCL18 directly promoted Siglec-15 expression. (D-E) Western blotting results revealed that CCL18 upregulated Siglec-15 expression through phosphorylation of the transcription factor STAT3. (F) Heatmap showing differentially expressed genes (DEGs) between BT549 TNBC cells with differentially expressed Siglec-15 groups, based on RNA sequencing data. (G-H) Bubble plots of Gene Ontology (GO) and Encyclopedia of Genes and Genomes (KEGG) functional enrichment analyses for DEGs in Siglec-15-manipulated BT549 TNBC cells (RNA sequencing data). (I) Heatmap of differentially expressed proteins in supernatants from Siglec-15-manipulated BT549 TNBC cells, identified via capillary reverse-phase liquid chromatography-tandem mass spectrometry (LC-MS/MS). (J-K) GO and KEGG functional enrichment analyses of differentially expressed proteins in supernatants from Siglec-15-manipulated BT549 TNBC cells (LC-MS/MS data). (L-M) qRT-PCR and ELISA assays characterized DcR3 expression patterns across TNBC cell groups. (N) Western blotting validated Siglec-15 promote DcR3 expression by activating the NF-κB pathway in BT549 TNBC cells. (*p < 0.05; **p < 0.01; ***p < 0.001; ****p < 0.0001).

### Siglec-15 modulates the expression of DcR3 via the NF-κB signaling pathway

To clarify the mechanism by which Siglec-15 triggers the recruitment and polarization of TAMs, RNA-seq and LC-MS/MS were performed on the RNA and cell supernatants of transfected BT549 cells. After we analyzed the RNA-seq, a total of 102 DEGs were identified. These DEGs were visualized using a volcano plot and a heatmap (Fig. 6F, Supplementary Fig. 4G). Through GO and KEGG enrichment analyses, it was found that Siglec-15 is involved in multiple tumor cell behaviors, including signal transduction, response to lipopolysaccharide, negative regulation of angiogenesis, and the JAK-STAT signaling pathway (Fig. 6G and H). Simultaneously, 89 DEGs of secreted proteins induced by Siglec-15 were selected (Fig. 6I, Supplementary Fig. 4H). GO and KEGG enrichment analyses were also conducted on these secreted protein DEGs. The results showed involvement in numerous signal transduction processes, such as cytokine - cytokine receptor interaction, extracellular matrix (ECM)-receptor interaction, and regulation of signaling receptor activity, as well as tumor cell behaviors, including the TGF-beta signaling pathway, positive regulation of the stress - activated MAPK cascade, and proteoglycans in cancer (Fig. 6J and K). Notably, the expression of TNFRSF6B (DcR3), a decoy receptor, was higher in both cell RNA and cell supernatants when Siglec-15 was overexpressed. Multiple studies have demonstrated that DcR3 can polarize M0 - type macrophages into M2 - type macrophages [[39], [40], [41]]. Subsequently, a lentivirus expression system using short hairpin RNA (shRNA) was used to knock down DcR3 in BT549 and MDA-MB-231 TNBC cells with either Siglec-15 overexpression or a vector control. The qRT-PCR and Elisa assay were carried out to evaluate the expression of DcR3. The results showed elevated DcR3 levels in the Siglec-15 overexpression group and decreased DcR3 levels in the DcR3 knockdown groups (Fig. 6L and M, Supplementary Fig. 4I). These findings indicated that Siglec-15 can regulate the expression of DcR3 at both the mRNA and protein levels. Previous research has revealed that the upregulation of DcR3 occurs through the nuclear factor-kappaB (NF-κB) activation pathway. Therefore, an inhibitor of the NF-κB pathway (QNZ) was added to BT549 overexpressing Siglec-15. Western blotting was used to detect the phosphorylation of NF-κB pathway proteins and the expression of DcR3. The results showed that with the inhibition of the NF-κB pathway, the expression of DcR3 also decreased, suggesting that Siglec-15 induces the expression of DcR3 through the NF-κB pathway (Fig. 6N).

### Siglec-15 induces the recruitment and polarization of TAMs through DcR3

In the blocking experiments, we knocked down DcR3 in TNBC cells overexpressing Siglec-15. Using qRT-PCR and flow cytometry analysis, we observed significant changes in the expression of M1/M2 macrophage biomarkers. In the group where DcR3 was knocked down, compared with the group overexpressing Siglec-15, the expression levels of certain M1 macrophage biomarkers (CD64, CD86, TNF-α) in co -cultured TAMs were elevated, while the expression levels of some M2 macrophage biomarkers (CCL2, CCL18, CD163) were reduced (Fig. 7A and B). In the recruitment assays, the number of M0 and M2 TAMs that migrated through the upper chamber was lower in the DcR3-knocked-down TCM group than in the Siglec-15 overexpression TCM group (Fig. 7C and D). These findings indicate that DcR3 can partially counteract the impact of Siglec-15 on the recruitment and polarization of TAMs. In addition, we collected the MCM from the co - culture systems of M2 type- CCL18⁺ TAMs with either Siglec-15 overexpression or DcR3 knockdown and added it to wild-type TNBC cells. We found that the proliferation, migration, and invasion abilities of TNBC cells induced by the MCM from the DcR3-knocked-down M2 type-CCL18⁺ TAMs were weaker than those induced by the MCM from the Siglec- 15-overexpressing M2 type-CCL18⁺ TAMs (Fig. 7E–G). This suggests that DcR3 can partially abolish the promoting effect of M2 type-CCL18⁺ TAMs on the malignant behaviors of TNBC cells. Finally, we verified the promoting effect of Siglec-15 on tumor growth in vivo (Fig. 7J and K).

**Fig. 7:**
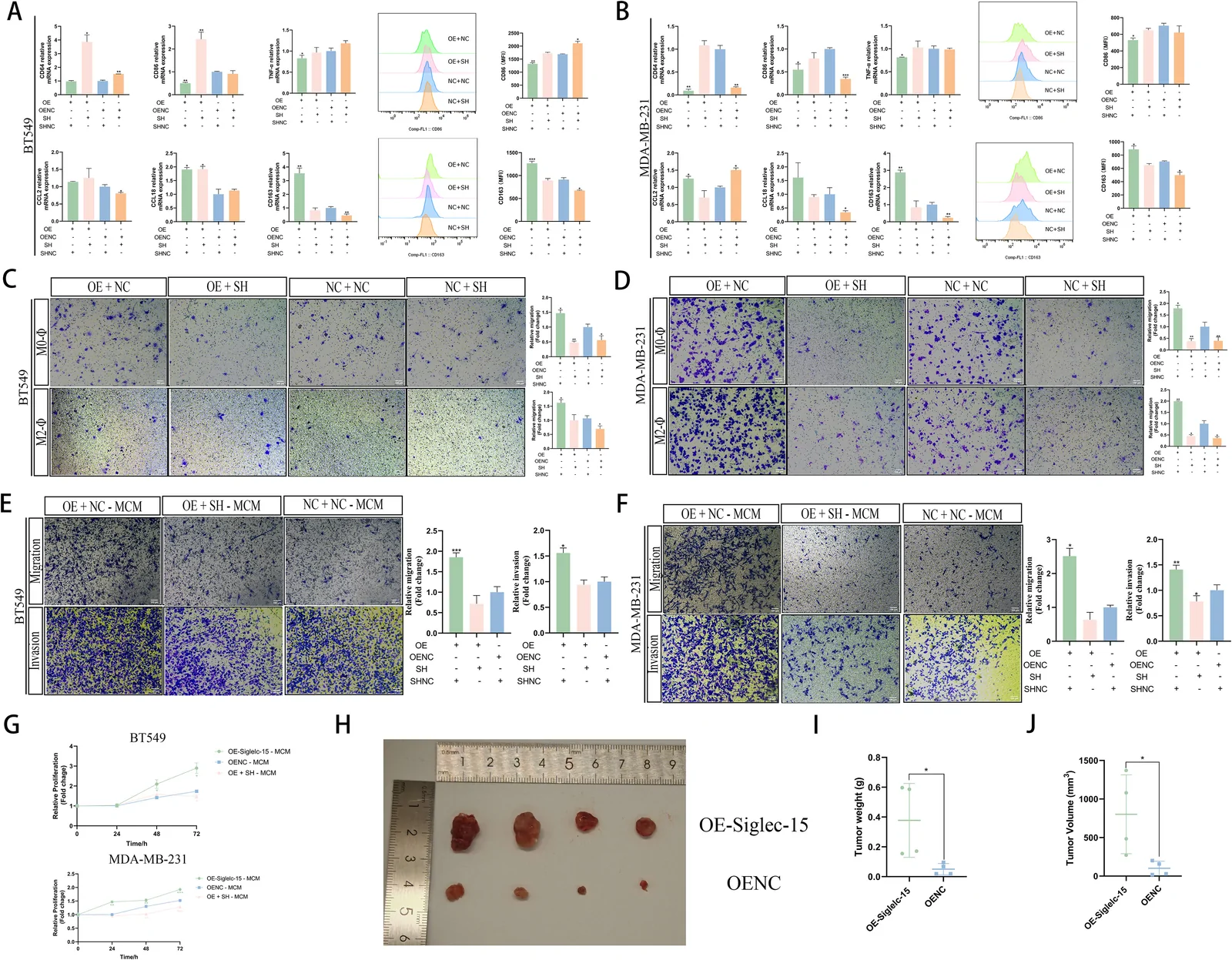
DcR3 partially reverses the effect of Siglec-15 on macrophages polarization and recruitment and tumor progression. (A-B) Quantitative real-time PCR (qRT-PCR) and flow cytometry analyses demonstrated that DcR3 partially reversed Siglec-15-mediated polarization of M0 macrophages toward M2 type-CCL18⁺ macrophages. (C-D) DcR3 mitigated Siglec-15-mediated recruitment of both M0 and M2 macrophages via transwell migration assays. (E-F) The conditional medium from co-culture system of M0-differentiated tumor-associated macrophages (TAMs) and MDA-MB-231, BT549 TNBC cells with differentially expressed genes (MCM) of knock-down DcR3 could inhibit the Siglec-15 overexpression-induced enhancement of TNBC cell migration and invasion, as determined by transwell assays. (G) CCK-8 viability assays showed MCM of knock-down DcR3 suppressed the proliferation of TNBC cells promoted by Siglec-15 overexpression. (H-J) Representative tumor images, along with measurements of tumor volume and weight, confirmed the tumor-promoting effects of Siglec-15 in vivo. (*p < 0.05; **p < 0.01; ***p < 0.001; ****p < 0.0001).

## Discussion

Breast cancer, a disease with high incidence and mortality rates, has drawn considerable attention over the past few decades. Among the various subtypes of breast cancer, TNBC exhibits the most aggressive and malignant phenotypes, for which the identification of reliable therapeutic gene targets remains an urgent research priority. With the rapid advancement of high-throughput sequencing technologies, an increasing number of novel prognostic biomarkers and therapeutic targets for TNBC have been identified. In this study, we employed scRNA-seq data, bulk transcription data and spital transcription data to systematically explore characteristic molecular alterations and key functional genes in TNBC. Specifically, we combined scRNA-seq and bulk transcriptomic datasets and applied the ssGSEA algorithm to evaluate the progressive status of TNBC tumor cells. A similar analytical strategy has been previously implemented in lung adenocarcinoma (LUAD) research to identify a UPP1 tumor cell subpopulation with prominent prognostic significance [47]. In this study, the T14 sub–cluster of TNBC tumor cells also demonstrated significant prognostic value. In addition to Siglec-15, the T14 subcluster is characterized by high expression of SPINK8, UGT2B11, UGT2B28, and SLC26A3. Functionally, SPINK8 is involved in the arachidonic acid metabolism and linked to poor prognosis in breast cancer [54]. Similarly, UGT2B11 and UGT2B28, key enzymes in steroid hormone and lipid metabolism, are highly expressed in prostate and breast cancer cells and promote lipid synthesis via the SREBP pathway [55,56]. Notably, SLC26A3 has been identified as a marker of neoadjuvant chemotherapy resistance in HER2-negative breast cancer [57]**.** Collectively, these markers suggest that the T14 subcluster is characterized by active metabolic reprogramming, which may contribute to its aggressive phenotype and adverse clinical outcomes. Accumulating evidences have demonstrated that metabolic reprogramming within the TME reshapes antitumor immunity and influences immunotherapy responses, targeting the metabolic vulnerabilities of T14‑like cells may offer a promising therapeutic avenue for TNBC [58]. We further analyzed its enrichment functions, transcriptional heterogeneity, and transcription factors (TFs) to gain a deeper understanding of its characteristics. In addition, machine learning algorithms have been extensively applied for prognostic prediction and biomarker screening in tumor research [[59], [60], [61]]. Accordingly, we screened prognostic marker genes specific to the T14 subcluster and constructed a prognostic model using 107 combinations derived from 11 machine learning algorithms. Model performance was comprehensively evaluated based on the C-index, and the obliqueRSF algorithm was ultimately selected as the optimal prognostic model with the highest predictive efficacy. Time-dependent ROC curve analysis and survival validation were further performed to verify the stability and reliability of the established model.

The TME, composed of non - cancerous cells, plays a crucial role in tumor progression [62]. In particular, immune cells within the TME have been proven to significantly influence tumor development [16,[63], [64], [65]]. We used the CellPhoneDB algorithm to analyze the interactions between tumor cells and immune cells. Among these interactions, the connections between tumor cells and macrophages were the most prominent, suggesting that macrophages may promote the progression of tumor cells. After analyzing TAMs, we identified six sub-clusters. Traditionally, M1 macrophages are considered anti-tumor, while M2 macrophages, known as TAMs, can promote the malignant behaviors of tumor cells in the TME [[66], [67], [68]]. Indeed, the clinical relevance of this dichotomy is underscored by observations that M1-like tumor infiltration correlates with improved chemotherapeutic outcomes across various malignancies [69]. We selected 19 M2 TAMs gene markers and 8 immune markers [47] to scored these sub-clusters and identified CCL18^+^ TAMs as the sub-cluster with the highest score. In Esophageal squamous cell carcinoma (ESCC), TAMs could secrete CCL18 to promote the tumor proliferation, leading to a poorer prognosis for patients [70]. In colorectal liver metastasis, MRC1^+^ CCL18^+^ M2-like macrophages have been found to possess immunosuppressive and metabolic activation features, and these cells can be targeted by effective neoadjuvant chemotherapy [71]. In our study, the expression of CCL18 was higher in M2 macrophages than in M0 macrophages derived from THP-1 cells. Additionally, in the spatial data, CCL18 and M2 marker genes (CCL2, CD163) exhibited similar expression patterns.

In this study, we employed immune cell infiltration analysis to explore the core gene in TNBC tumor cells that can influence the expression of CCL18⁺ TAMs. The results showed that Siglec-15 was positively correlated with M0/M2 macrophages and negatively correlated with M1 macrophages. Survival analysis indicated that high expression of Siglec-15 was associated with poorer survival outcomes. Siglec-15 has been confirmed as an immune suppressor and serves as a target for cancer immunotherapy, with upregulation observed in multiple cancer types [11,72,73]. In breast cancer bone metastasis, inhibiting the Siglec-15/sialic acid axis can treat bone metastasis and prevent the spread of tumor cells from bone to other organs [13]. Similarly, it has been found that Siglec-15 can bind to Toll-like receptor 2 (TLR2) to inhibit the activation of naïve CD8⁺ T cells. Targeting Siglec-15 can reduce the incidence of secondary metastases and improve prognosis [14]. In our investigation, we detected high expression of Siglec-15 in TNBC patients. Then, through transwell co-culture assays, we determined the impact of Siglec-15 on macrophage recruitment and polarization. In TNBC cells, Siglec-15 promotes the recruitment of M0 and M2 macrophages and the polarization of M2 type-CCL18⁺ TAMs. Moreover, the CM collected form the co-culture system of M2 type-CCL18⁺ TAMs could enhance the proliferation, migration, and invasion abilities of TNBC cells. Similar to findings in LUAD and colorectal cancer, TAMs co-cultured with tumor cells can promote the malignant behaviors of tumor cells [65,74], and Siglec-15 can promote tumor growth in vivo.

Subsequently, we investigated whether M2 TAMs could regulate Siglec-15 expression. In our experiment, when the CM from M0/M2 was added to wild-type TNBC cells, the expression of Siglec-15 was elevated in the group treated with M2 CM. Moreover, the addition of CCL18 further enhanced the expression of Siglec-15

These results suggest that the secretion of CCL18 could promote the expression of Siglec-15. Therefore, M2 type-CCL18⁺ TAMs not only directly promote tumor cell progression but also upregulate Siglec-15 expression, indirectly facilitating tumor proliferation, migration, and invasion in TNBC. Through a comprehensive analysis of the hTFtargets and JASPAR databases, we predicted the potential TFs of Siglec-15 in breast cancer. STAT3,as a transcription factor, has been reported to be regulated by CCL18 in intrahepatic cholangiocarcinoma, oral cancer, and non-small cell lung cancer [19,30,31]. However, such regulatory changes in breast cancer have been rarely reported. Notably, recent studies have established that membrane-associated molecules in TNBC, particularly CD44v5, can mediate crosstalk with TAMs by activating the IL-4R/STAT3 and IL-4/IL-4Rα/STAT6/SAM68 axis, thereby driving M2 polarization and tumor progression [75,76]. These findings indicate that STAT3 and STAT6 serve as central signaling hub that bridge tumor membrane proteins and the tumor microenvironment.

In the present study, we extend this regulatory paradigm, demonstrating that Siglec-15, another membrane-associated immune checkpoint, trigger the NF-κB/DcR3/CCL18 cascade to ultimately promote STAT3 phosphorylation, thereby forming a positive feed-forward loop that sustains CCL18⁺ TAM polarization and accelerates TNBC malignant progression.

To analyze the specific mechanism by which Siglec-15 modulated macrophage polarization and TNBC progression, we performed transcriptome sequencing and secretory proteome profiling of TNBC cell supernatants. Intriguingly, both RNA-seq and mass spectrometry analyses consistently identified significantly upregulated DcR3 expression under Siglec-15 overexpression, indicating that Siglec-15 promotes the expression and secretion of Decoy receptor 3 (DcR3). DcR3, a member of the tumor necrosis factor receptor (TNFR) superfamily member 6b (TNFRSF6B), is highly expressed under pathological conditions [77]. As a special receptor, DcR3 could bind to three ligands: FasL (CD95L/TNFSF6) [78], LIGHT (CD258/TNFSF14) [79], and TNF-like molecule 1A (TL1A/VEGI/TNFSF15) [80] to inhibit tumor cells death. Previous research has shown that DcR3 can polarize macrophages towards the M2 phenotype [40], which further facilitates tumor malignant progression [39]. In papillary thyroid carcinoma, the high expression of DcR3 was positively related with M2 TAMs and lymphatic invasion [41]. Consistent with these findings, our functional validation revealed that reducing DcR3 expression partially reversed the effect of Siglec-15 on the recruitment of M0 and M2 macrophages and CCL18⁺ macrophage polarization, and DcR3 can inhibit the enhancement of the malignant behaviors of CCL18⁺ macrophages.

Furthermore, we were interested in the reason why Siglec-15 induces DcR3 expression. In pancreatic adenocarcinoma, inhibiting the PI3K-Akt and NF-κB signaling pathways reduces DcR3 expression [37]. In nasopharyngeal carcinoma, NF-κB signaling pathway inhibitors have a stronger inhibitory effect on DcR3 secretion than PI3K-Akt signaling pathway inhibitors [38]. In our study, adding an NF-κB signaling pathway inhibitor decreased the high expression of DcR3 induced by Siglec-15 in TNBC cells, further validating that DcR3 expression is regulated by the NF-κB signaling pathway.

Despite our comprehensive analyses in this study, there are many limitations. First, stromal cells were excluded during the analysis of single-cell RNA sequencing analysis, which limits a holistic understanding of the entire TME landscape. Given that CAFs and other stromal components are critical modulators of immune cell function and infiltration, future studies incorporating stromal compartments will help refine the TME regulatory network [81,82]. Second, although our results strongly support the existence of the CCL18/STAT3/Siglec-15 regulatory axis, additional causal evidence from ChIP-qPCR, luciferase reporter assays, and specific pharmacological or genetic inhibition of STAT3 will further solidify this mechanistic model. Furthermore, the mechanism by which Siglec-15 signaling directly activates the NF-κB/DcR3 axis remains unclear. As a membrane protein, Siglec-15 primarily functions through intracellular ITIM-like motifs. Future experiments using Siglec-15 intracellular tail truncation mutants will help verify the direct regulatory relationship between Siglec-15 signaling and the NF-κB/DcR3 axis. In addition, there are many limitations in the in vivo experiments. The current xenograft study only confirmed the pro-tumor effect of Siglec-15 at the macroscopic level; however, TAM infiltration was not evaluated by IHC staining, and only Siglec-15-overexpression and vector control groups were established without the application of the inhibitors of STAT3 or NF-κB pathway. Future in vivo investigations with larger animal cohorts, specific blockade of the STAT3/NF-κB pathway, and comprehensive IHC detection of macrophage polarization markers (CD86, CD163, and CD206), together with multiplexed cytokine profiling, will further validate the translational potential of the Siglec-15-mediated feed-forward regulatory loop in TNBC.

In conclusion, this study provides new insights into the TME of TNBC. For the first time, we discovered that high Siglec-15 expression can establish a positive feed-forward regulatory loop between TNBC cells and TAMs through the NF-κB/DcR3/CCL18/STAT3 axis, which ultimately facilitates TNBC progression (Fig. 8). Our findings deepen the current understanding of TME remodeling in TNBC and identify Siglec-15 as a promising therapeutic biomarker, providing valuable clues for the development of targeted therapeutic strategies for TNBC.

**Fig. 8:**
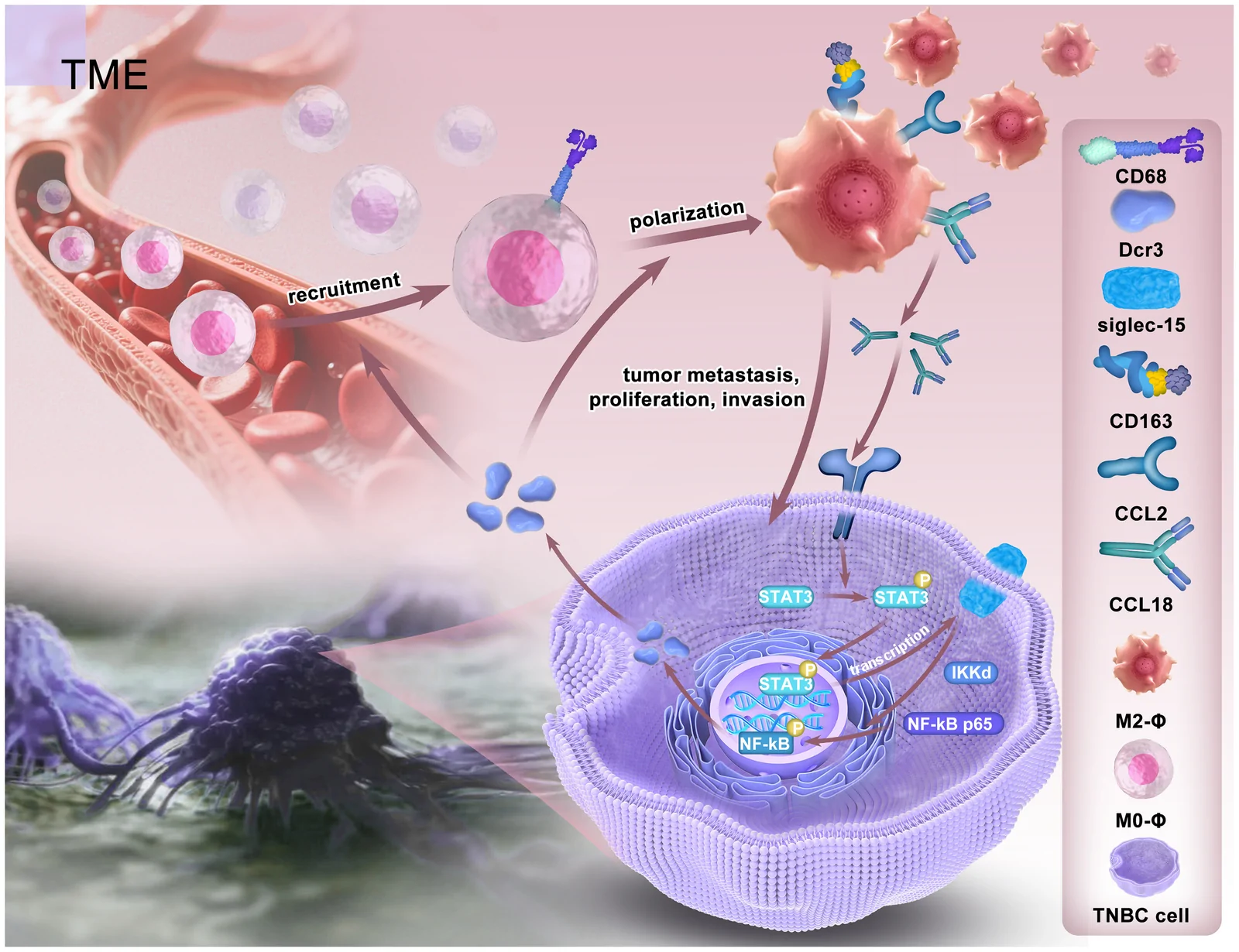
Schematic model illustrating Siglec-15-mediated promotion of a feed-forward loop between TNBC cells and macrophages via the NF-κB/DcR3/CCL18/STAT3 axis.

## Consent for publication

All authors have agreed to the publication of this manuscript.

## Ethics statement

All protocols and experiments involving human subjects were approved by the Institutional Review Board of Qingdao Central Hospital, University of Health and Rehabilitation Sciences (approval number: KY202504801). All participants provided written informed consent prior to their enrollment in this study. All protocols and experiments about mice were approved by Institutional Review Board of the Qingdao University of Science and technology (QKDLL-2025-033)

## Funding information

This study was supported by the Qingdao Key Health Discipline Development Fund, Qingdao Medical and Health Research Project Fund (2024-WJKY072), 2026 Shandong Provincial Medical and Health Science and Technology Project (General Program, Grant No. 202604010913), and Wu Jieping Medical Fund (320.6750.2022-19-37).

## CRediT authorship contribution statement

**Sizhang Wang:** Methodology, Investigation, Funding acquisition. **Hua Qi Wang:** Project administration, Funding acquisition, Formal analysis, Conceptualization. **Hui Zhao:** Software, Investigation, Funding acquisition. **Ruixia Zhao:** Resources, Project administration, Investigation, Funding acquisition. **Peng Zhao:** Writing – original draft, Visualization, Software, Investigation. **Qiang Mu:** Validation, Supervision, Investigation, Funding acquisition.

## Declaration of competing interest

The authors declare that they have no competing interests.

## Data Availability

The primary data used in this study were from the GEO, TCGA, and Metabric databases. The microarray GSE161529, GSE58812 and GSE218951 data were from the GEO database. For additional information, please feel free to get in touch with the corresponding author.
