# Supplementary material for: Siglec–15 promotes a feed-forward loop between TNBC cells and macrophages via the NF-κB/DcR3/CCL18/STAT3 axis
Source: Transl Oncol. 2026 Sep 18;73:103032. doi: 10.1016/j.tranon.2026.103032 (PMC13625897; doi:10.1016/j.tranon.2026.103032)
Supplement: Supplementary file 2 [file mmc1.doc]

Supplementary Materials for Siglec–15 promotes a feed-forward loop between TNBC cells and macrophages via the NF-κB/DcR3/CCL18/STAT3 axis

Authors: Sizhang Wang^a^, Hua Qi Wang^a^, Ying Tian^a^, Ruixia Zhao^*b^, Peng Zhao^*c^, Qiang Mu^*d^

Affiliation: a Qingdao Medical College of Qingdao University, 266042, Qingdao, Shandong, China.

b Department of Pathology, Qingdao Central Hospital, University of Health and Rehabilitation Sciences (Qingdao Central Hospital), 266042, Qingdao, Shandong, China

c Department of Molecular Pathology, Qingdao Central Hospital, University of Health and Rehabilitation Sciences (Qingdao Central Hospital), 266042, Qingdao, Shandong, China

d the First Department of Breast Surgery, Qingdao Central Hospital, University of Health and Rehabilitation Sciences (Qingdao Central Hospital), 266042, Qingdao, Shandong, China

* Correspondence: 17805325589@163.com(R, Zhao), zhaopeng@uhrs.edu.cn(P. Zhao), [muqiang@qdu.edu.cn](mailto:muqiang@qdu.edu.cn) (Q. Mu).

This file includes:

Supplementary Figure captions

Figures S1 to S3

Supplementary Table captions

Supplementary Fig.1. Procession of scRNA-seq of TNBC cells. (A) UMAP visualization of 32 clusters identified in samples from 8 TNBC patients and 13 normal patients. (B-D) UMAP plot highlighting the expression of epithelial cells marker gene(EPCAM,KRT8,KRT19) across 8 TNBC patients and 13 normal patients. (E-F) Heatmap illustrating copy number variation changes comparing normal individuals and TNBC patients. (G-I) UMAP projection showing the distribution of immune cells marker gene(CD45,CD3D,CD68) specifically in 8 TNBC patients.

Supplementary Fig.2. scRNA-seq analysis of TNBC cells. (A) UMAP plot depicting 31 clusters of tumor cells and immune cells in 8 TNBC patients. (B) Violin plots displaying the expression patterns of distinct marker genes across the 31 clusters. (C) Visualization of the expression pattern of marker genes corresponding 5 major clusters. (D-E) Scatter plots illustrating tumor cell differentiation trajectories inferred via Monocle2 analysis. (F) Box plots presenting CytoTRACE analysis results for predicting the differentiation status of tumor cells.

Supplementary Fig.3. The analysis of TAMs of scRNA-seq (A) Bar plots showing the top30 genes most strongly correlated with tumor cell differentiation status, as determined by CytoTRACE analysis. (B) Venn plots identifying the 437 overlapping genes between cluster marker genes and differentially expressed genes (DEGs) comparing TNBC patients and normal controls. (C) UMAP plot showing the expression of Siglec-15 across TNBC tumor cells. (D) UMAP visualization identifying 7 macrophages clusters within TNBC tumor cells. (E-F). Dot plots showing the expression levels of immune suppression genes and M2 polarization signatures across the 6 macrophage sub-clusters in TNBC tumor cells. (G) Heatmap quantifying cell–cell interaction frequencies between tumor cell subclusters and macrophage sub-clusters, as determined by CellPhone DB analysis. (H) Box plots presenting prediction of the differentiation status of the macrophages populations by CytoTRACE analysis. (I) Bar plots highlighting top30 genes, which have most correlation with the differentiation of the macrophages using CytoTRACE analysis.

Supplementary Fig.4. Procession of spital transcription data of TNBC cells and multi-omics analysis of TNBC cells. (A-B) Unsupervised clustering of all cells into 9 distinct clusters across two TNBC tissue sections. (C-E) Scatter plots displaying no significant correlation between Siglec-15 expression and M0/M1/M2 macrophage polarization states. (F) Kaplan–Meier survival analysis indicating that higher Siglec-15 expression correlates with improved overall survival in TNBC patients from Metabric cohort. (G) Volcano plot visualizing 107 significantly differentially expressed genes (DEGs) between BT549 TNBC cells with differentially expressed Siglec-15 groups, based on RNA sequencing data. (H) Volcano plot of 89 significant differentially expressed proteins in supernatants from Siglec-15-manipulated BT549 TNBC cells, identified via capillary reverse-phase liquid chromatography-tandem mass spectrometry (LC-MS/MS). (I). Quantitative real-time PCR (qRT-PCR) demonstrated that elevated Siglec-15 expression in TNBC cells with stable Siglec-15 overexpression.

Fig. S1.

Fig. S2.

Fig. S3.

Fig.S4.

**Table captions**

Table S1 The data 149 prognostic genes by KM survival analysis.

Table S2 All primer sequences.

Table S3 The data of differentially expressed gene between BT549 TNBC cells with differentially expressed Siglec-15 groups, based on RNA sequencing data.

Table S4 The data of differentially expressed proteins in supernatants from Siglec-15-manipulated BT549 TNBC cells, identified via capillary reverse-phase liquid chromatography-tandem mass spectrometry (LC-MS/MS).
