# Supplementary material for: Siglec–15 promotes a feed-forward loop between TNBC cells and macrophages via the NF-κB/DcR3/CCL18/STAT3 axis
Source: Transl Oncol. 2026 Sep 18;73:103032. doi: 10.1016/j.tranon.2026.103032 (PMC13625897; doi:10.1016/j.tranon.2026.103032)
Supplement: Supplementary file 3 [file mmc3.doc]

Table S1 All primer sequences.

| Names | Sequence | Tm |
| --- | --- | --- |
| H-Siglec15-F | TTTGAGCCAGATGAACCCCC | 59.96 |
| H-Siglec15-R | CAGGGAGCTCCGAAATGGTT | 60.04 |
| H-CCL2-F | AAGATCTCAGTGCAGAGGCTCG | 62.10 |
| H-CCL2-R | CACAGATCTCCTTGGCCACAA | 60.27 |
| H-CD163-F | CTTGGGACTTGGACGATGCT | 60.04 |
| H-CD163-R | GGTATCTTAAAGGCTCACTGGGT | 59.80 |
| H-CD86-F | GCTTTGCTTCTCTGCTGCTG | 60.11 |
| H-CD86-R | GGCAGGTCTGCAGTCTCATT | 60.04 |
| H-TNFRSF6B-F | CCACTACACGCAGTTCTGGA | 59.68 |
| H-TNFRSF6B-R | GTGCTCCAAGCAGAAACCAG | 59.40 |
| H-CD68-F | CTTCTCTCATTCCCCTATGGACA | 59.03 |
| H-CD68-R | GAAGGACACATTGTACTCCACC | 58.66 |
| H-FCGR1A-F | GCATGGGAAAGCATCGCTAC | 59.69 |
| H-FCGR1A-R | GCAAGAGCAACTTTGTTTCACA | 58.49 |
| H- CCL18 -F | CACTCTGACCACTTCTCTGC | 57.92 |
| H- CCL18 -R | TGCCAGGAGGTATAGACGAG | 58.02 |
| H-MRC1-F | GGACGTGGCTGTGGATAAAT | 57.96 |
| H-MRC1-R | ACCCAGAAGACGCATGTAAAG | 58.30 |
| H-GAPDH-F | AGGTCGGAGTCAACGGATTTGG | 59 |
| H-GAPDH-R | TGCCATGGGTGGAATCATATTGG | 60 |
| H-TNF-α-F | ATGTTGTAGCAAACCCTCAAGC | 59.44 |
| H-TNF-α-R | TGTGGGTGAGGAGCACAT | 58.09 |
